# The influence of potassium post-deposition treatments on grain boundaries and surfaces of Cu(In,Ga)Se_2_ absorbers

**DOI:** 10.1038/s41598-026-55190-9

**Published:** 2026-06-03

**Authors:** Evandro Martin Lanzoni, Omar Ramírez, Muhammad Uzair Farooq, Oana Cojocaru-Mirédin, Jean-Nicolas Audinot, Tom Wirtz, Susanne Siebentritt, Alex Redinger

**Affiliations:** 1 Department of Physics and Materials Science, University of Luxembourg, Luxembourg, Luxembourg; 2 INATECH, Albert-Ludwigs Universität, 79110 Freiburg, Germany; 3 Advanced Instrumentation for Nano-Analytics (AINA), Luxembourg Institute of Science and Technology (LIST), L-4362 Esch-sur-Alzette, Luxembourg

**Keywords:** Alkali post-deposition treatment, Copper Indium Gallium Selenide, Solar cells, Grain boundary, Energy science and technology, Materials science, Physics

## Abstract

Alkali metal post-deposition treatments (PDTs) have played a pivotal role in the development of high-efficiency Cu(In,Ga)Se_2_ thin film solar cells. The PDTs impact the near-surface region of the absorbers, the grain boundaries, and the bulk. Since Cu(In,Ga)Se_2_ solar cells are polycrystalline, the investigations turn out to be very challenging as each grain/grain boundary is affected slightly differently by the PDTs. Therefore, we synthesized Cu(In,Ga)Se_2_ absorbers on microcrystalline GaAs substrates to form millimeter-sized epitaxial domains with well-defined grain boundaries. This model system allows us to study the same surface orientation and grain boundary before and after PDTs. We find strong preferential (112) facets and Cu depletion on the surface, with the grain boundaries exhibiting very similar Cu-depleted compositions. After K deposition and annealing, Cu is pushed into the absorber, leaving a K-rich compound at the near-surface. Furthermore, we find different diffusion rates of K through the grain interior and via the grain boundaries. The exchange of Cu with K at the surface also occurs at the grain boundaries as measured with atom probe tomography. High-resolution KPFM measurements performed on the same grain boundary before and after PDT treatment show almost negligible grain boundary band bending. Cu-depleted K-rich area at the surface and grain boundaries exhibit very similar compositions, which gives rise to small band bending values. Our results give an unprecedented view of the mechanisms that occur at the surface, grain boundaries, and the bulk of CIGSe solar cells, paving the way to further develop the materials and improve the power conversion efficiency.

**Supplementary Information:**

The online version contains supplementary material available at 10.1038/s41598-026-55190-9.

## Introduction

Cu(In,Ga)Se_2_ (CIGSe) thin-film solar cells are among the highest performing thin-film-based solar cell technologies that have been developed over the past several decades. They encompass efficiencies well beyond 20%^1,2^ in the laboratory and have proven excellent long-term stability and performance under standardized test conditions, as well as in operating modules^3–5^. Recent advances in this technology can be attributed to the development of post-deposition treatments (PDTs) with alkali fluorides, which allow higher open-circuit voltages and thinner buffer layers^1,2,6,7^. To further develop CIGSe thin-film solar cells, the impact of PDTs needs to be understood as adequate as possible.

However, the interaction of the alkali-metal fluorides with the surface of CIGSe is extremely complex. Since the CIGSe solar cell is polycrystalline, its surface consists of micrometer-sized grains with different surface terminations. The same holds true for grain boundaries (GBs), which exhibit different densities of structural defects due to different orientations of the surrounding grains. It was shown that the properties of the CIGSe surface, including the density of defect states and the surface work function^8,9^, as well as the coverage of the subsequently deposited buffer layer^10–12^, vary according to the crystallographic facet. Furthermore, regardless of whether Na, K, Rb, or Cs were used during the PDTs, an accumulation of alkali metals at the GBs was measured by atom probe tomography (APT)^13–16^. The exact mechanism dictating how the alkali metals segregate at the GB is currently not clear. In addition, modifications to the optoelectronic properties at the GBs are not conclusive. Cathodoluminescence measurements show that the recombination velocity is independent of the PDTs^17^, suggesting that the passivation of the GB defects is not the dominant effect of the PDTs. However, these results also did not show an improvement in power conversion efficiency due to the PDTs. However, reports suggesting that alkali elements passivate the GBs can be found in the literature, where Kelvin probe force microscopy (KPFM) measurements showed a reduction in the average band bending at the GB after PDT^18^. The Cu content of the CIGSe absorbers is extremely important, and it has been shown that high-efficiency CIGSe absorbers exhibit mainly Cu-depleted GBs^19^. This intrinsic Cu depletion is most likely a direct consequence of Cu-poor growth conditions used to make high-efficiency CIGSe solar cells. In addition to the mostly Cu-depleted GBs, the surface of Cu-poor CIGSe is also Cu-depleted, which is also modified by PDTs^6,20,21^.

Given the facet-dependent properties, the impact of the PDTs needs to be studied, in the ideal case, on exactly the same grain, exhibiting a well-defined surface orientation. Similarly, the same GB should be analyzed to understand how the alkali metals impact the GB properties. At present, reports in the literature are limited to statistical averages over many grains and GBs.

The beneficial effects of alkali elements are well recognized in the literature. The PDT processes are done almost exclusively by evaporating alkali-metal fluorides instead of pure metals. The impact of fluorine on the bulk CIGSe properties is not clear. At the surface, the treatment with alkali fluoride PDT leads to the formation of GaF$\vphantom{0}_3$, which can be removed via H$\vphantom{0}_2$O or NH$\vphantom{0}_4$OH cleaning^12^. This already shows that fluorine also has an impact on the surface composition of CIGSe, although in a more indirect way. In secondary ion mass spectrometry (SIMS), small amounts of F can also be found deeper in the bulk, even after cleaning with NH$\vphantom{0}_4$OH^21^. The impact of this very small amount is at present completely unclear.

In addition to the facet-dependent properties, the samples are often exposed to air and are consequently oxidized. The formation of $\textrm{Rb}_2$ $\textrm{SeO}_3$ has been reported together with $\mathrm{In(OH)}_3$, $\textrm{GaO}_x$, In-Se-O compounds^22^. Although most of these compounds can be removed subsequently via chemical etching, they all influence the surface composition and, thereby, potentially have an impact on the final device performance.

The essence of the literature review shows that understanding PDT treatments is hampered by the polycrystallinity of the material, the additional fluoride evaporated during PDT, and the reaction with oxygen and moisture before the buffer layer deposition. Therefore, it is important to eliminate most of the complications discussed above and design samples and measurement protocols that allow the study of the real impact of alkali metals only. In addition, the effect of oxidation and moisture should be removed, and well-defined crystallographic orientations should be used.

In this study, large macroscopic GBs were achieved by synthesizing CIGSe samples grown on multicrystalline GaAs substrates, which exhibited grain sizes in the millimeter range. Thereby, well-defined and well-separated GBs and surface orientations are formed, which could be analyzed before and after PDTs. PDTs were performed by evaporating K from alkali metal dispensers under an ultra-high vacuum (UHV), thereby eliminating any influence of fluorine. Finally, the growth and characterization were performed without measurable surface contamination, which allowed to study the impact of alkali elements without taking into account reactions with oxygen or moisture.

High-resolution Kelvin Probe Force Microscopy (KPFM) and X-ray Photoelectron Spectroscopy (XPS) measurements before and after PDT were used to correlate workfunction variations with changes in the surface composition. APT measurements carried out on GBs before and after PDT were used to better understand the modification in composition and possible dopant segregation at the GBs with respect to the near-surface region. In addition, high-resolution 3d SIMS mapping linked the measurements carried out at the surface to those measured in the bulk. Finally, the AFM tip was used for controlled removal of material from the near-surface region of the CIGSe and to probe GB properties in the bulk of the crystal.

The results show that the diffusion and incorporation of K are accompanied by Cu migration into the bulk. This process occurs on the surface and the GBs in a very similar manner. On the surface, an almost one-to-one exchange of Cu with K was observed, whereas on the GBs, the exchange was incomplete. SIMS analysis allowed quantifying the diffusion rate of K into the bulk and at the GBs. The diffusion coefficient is three times higher at the GBs than in the bulk, and a strongly asymmetric profile from top to bottom of the layer was found. The KPFM measurements at exactly the same GBs before and after PDT demonstrated that the GB band bending at the surface is negligible in both cases. This is a direct consequence of the similarity between the grains and GBs, which have very similar compositions. AFM tomography measurements allowed probing GB band bending in the bulk of the material. Band bending values are higher than at the surface, and for the limited set of measurements, a predominant downward band bending with a surface charge at the GB of approximately N$\vphantom{0}_\textrm{D}$=3.5*·* 10$\vphantom{0}^{10} \pm$ 1.2*·* 10$\vphantom{0}^{10}$ charges/$cm^{2}$ was deduced.

## Results

**Fig. 1:**
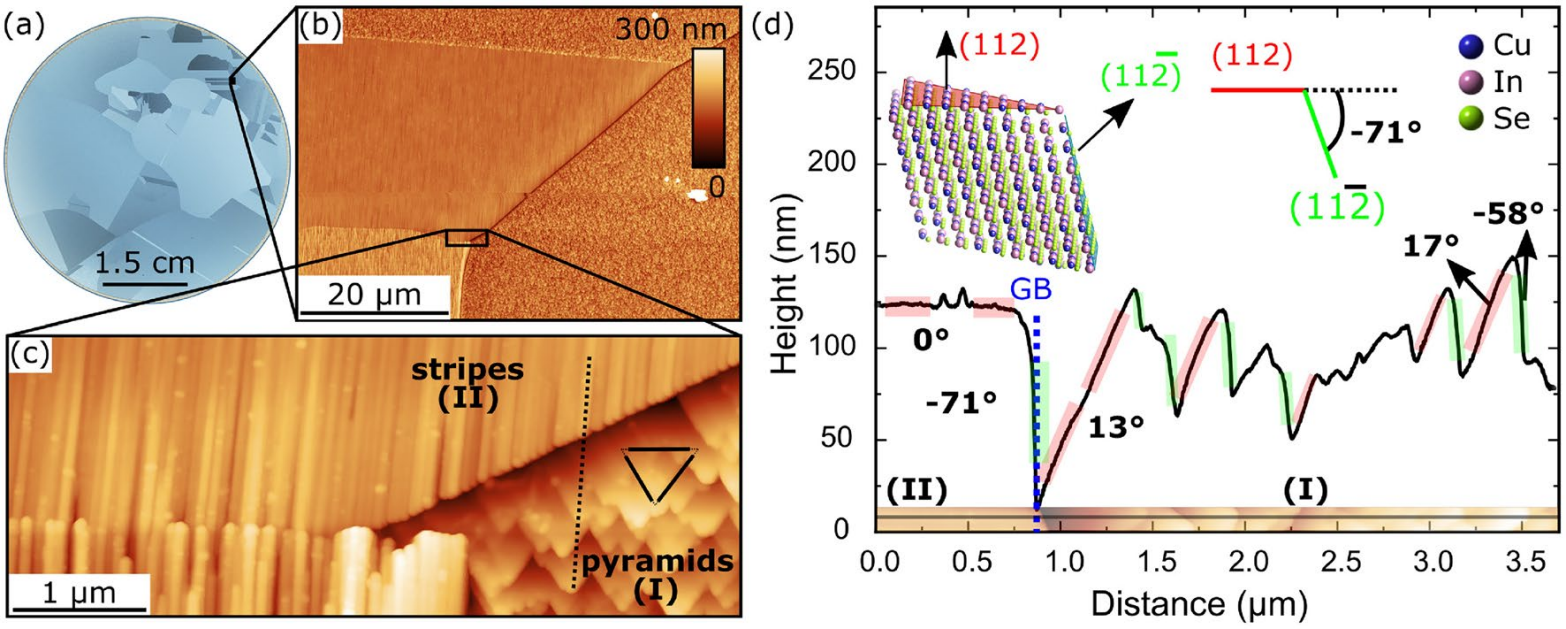
(**a**) Photograph of the GaAs multi-crystalline wafer coated with 500 nm of CIGSe. (**b**) AFM image of the highlighted region in (**a**). (**c** )High-resolution AFM topography image of the region highlighted in (**b**). (**d**) Line profile shown in (**d**) together with a ball model (perpendicular to the sample surface) that explains the different orientation visible in (**d**).

Figure 1a shows a picture of one of the two-inch multicrystalline GaAs wafers used as substrates for the growth of the epitaxial CIGSe layers. In the image, well-defined regions with distinguishable color contrast can be seen. Each of these regions consists of a slightly different crystallographic orientation. The subsequent epitaxial growth of CIGSe on such substrates then led to unique samples with millimeter-sized grains and distinct out-of-plane orientations imposed by the underlying substrate. Most interesting, it led to the formation of GBs between the different regions, which were sometimes several centimeters in length. In this context, the epitaxial samples were similar to the polycrystalline samples used in the production of CIGSe solar cells. The main difference was that the size of the grains and GBs was increased by a factor of approximately 1000 (from *μ*m to mm). This increase in zoom-multi-crystallinity of the GaAs substrate led to the growth of CIGSe films with well-defined surface orientations and macroscopic GBs, as illustrated in the AFM images in Fig. 1b.

Electron Backscatter Diffraction (EBSD) image quality maps of the GaAs substrate are shown in Fig. S1. For all investigated regions, small deviations in grain orientation from the main $(111)_{cub}$ wafer orientation were found. All GBs were low-angle GBs with misorientations smaller than 10 degrees. The investigated samples in this paper are therefore limited to low-angle GBs, meaning they are composed of an array of dislocations with the spacing between them being inversely proportional to the misorientation angle^23^.

Despite the small deviations from the main GaAs $(111)_{cub}$ orientation, the topography of the CIGSe layer is strongly affected by the underneath substrate, and regions with very different surface morphologies were observed, as shown in the AFM images of Fig. 1b, c. In Fig. 1c, which is a zoom-in of Fig. 1b, a stripe-like morphology was identified (region (II)), which at first sight was similar to the one of CIGSe grown on GaAs $(001)_{cub}$^24–27^. However, the region was very flat with a root mean square roughness of only 10 nm, which was not the case for the CIGSe layer grown on GaAs $(001)_{cub}$.

From EBSD, we identified the out-of-plane orientation of the GaAs substrate to be close to $(111)_{cub}$, which translates into a $(112)_{tet}$ direction for tetragonal CIGSe. Therefore, the CIGSe absorbers grow in an out-of-plane $(112)_{tet}$ direction, leading to very flat films, as already reported by Rockett^28^.

The second type of surface morphology, illustrated in Fig. 1c as region (I), displayed a tilted pyramid-like structure. The angles between the edges of the most prominent facet measured 60$\vphantom{0}^{\circ }$, as depicted for one of the pyramids. A close inspection of the triangles revealed that the edges were blunt, that is, the triangles were, in fact, hexagons with two different edge lengths. According to Rockett^28^, these correspond to the anion- and cation-terminated step edges, which exhibit different surface energetics. In that sense, both morphologies shown in Fig. 1b, c were $\{112\}_{tet}$ terminated, but the GB and the deviations from the $(111)_{cub}$ plane of the GaAs led to a tilting of the low-energy planes against each other. This can be corroborated by analyzing the two different regions of the CIGSe in more detail.

A line profile highlighted by a dashed line in Fig. 1c was analyzed next. The different regions are labeled (I) for the pyramid-like structure and (II) for the stripe-like structure. The angles between two different planes on pyramid-shaped structures were first analyzed. This surface feature was chosen since here the facets were quite extended and thereby tip-related smearing-out effects could be minimized. The indicated angles in Fig. 1d are all measured counterclockwise with respect to the horizontal. The angle between the two highlighted planes on the right side of Fig. 1d corresponds to 75 $\vphantom{0}^{\circ }$ ($17^\circ$+$58^\circ$), which is reasonably close to the expected angle between the $(11\bar{2})_{tet}$ and $(112)_{tet}$ planes^25^. All other planes, closer to the GB, also exhibited similar angles, as indicated by the two different shadings in the line profile. This meant that the two dominant planes had angles compatible with two different $\{112\}_{tet}$ planes. However, some were tilted with respect to the normal direction of the wafer, which was $(111)_{cub}$.

In the stripe-like region (labeled (II) in Fig. 1c, d), the ridges were all parallel to the horizontal plane, which was equal to a $\{112\}_{tet}$ terminated CIGSe surface, as discussed earlier. At the GB (see the blue dashed line in Fig. 1d), the angle was again close to the expected angle of 71$\vphantom{0}^{\circ }$ (cation and anion terminated $\{112\}_{tet}$ planes). However, this angle was also close to the maximum angle that can be measured with an AFM tip (half cone angle: 10$\vphantom{0}^{\circ }$).

From this discussion, it follows that both morphologies were made up of facets that were $\{112\}_{tet}$ terminated. In this case, both $\{112\}_{tet}$ terminations were tilted approximately 13$\vphantom{0}^{\circ }$ at the GB.

**Fig. 2:**
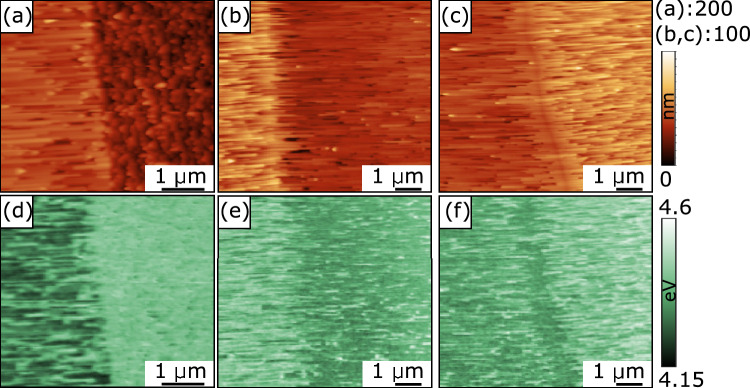
Three different GBs of the multicrystalline sample with 200 nm CIGSe. (**a**–**c**) topography, (**d**–**f**) corresponding workfunction map.

After the identification of the film’s crystallographic orientations, KPFM measurements were carried out to measure the local variations in workfunction. All samples presented in the following were never exposed to ambient conditions. The samples were transferred from the metal-organic vapor phase epitaxy (MOVPE) reactor through $\textrm{N}_2$-filled gloveboxes and then to the UHV KPFM via an $\textrm{N}_2$-filled transfer system. Figure 2 shows topography and workfunction maps of three different regions on the multicrystalline CIGSe wafer, where a single GB was observed in each image. A photo of the wafer and the respective positions of the GBs can be found in Fig. S2. In analogy to Fig. 1, the same two types of morphologies, namely stripes and pyramids, were found. The pyramid-like structures were approximately two times rougher than the stripe-like regions, as shown in Fig. 2a–c. The workfunction maps presented in Fig. 2d–f showed that the different regions exhibited different values of the workfunction. The pyramid-like structures in Fig. 2a on the right side of the image, exhibited an average workfunction of 4.4 eV ± 0.1 eV. As discussed previously, this workfunction corresponded to the $\{112\}_{tet}$ facet. The unambiguous assignment was possible because of the characteristic shape of the facets. As discussed before, the stripe-like regions also consist of $(112)_{tet}$ surfaces, which would translate into no workfunction contrast as the surface termination is identical. In fact, some parts of the stripe-like pattern had the same workfunction as the pyramids (see a zoom-in in Fig. S3). The rest of the stripes exhibited workfunction values that were on average 150 meV lower than the $\{112\}_{tet}$ facets. The angle between the two regions is 30-40 degrees, close to the expected value of the facet $\{001\}_{tet}$^26^. In analogy to the previous report^26^, the low workfunction regions were assigned to $\{001\}_{tet}$ facets.

Figure 2b, e shows an example of two stripe-like regions separated by a GB. The left part of the workfunction map in Fig. 2e exhibited an average value of 4.4 eV, consistent with a $\{112\}_{tet}$ surface termination. The right part showed a reduction in the workfunction in the vicinity of the GB (*≈* 50 meV), followed by a gradual recovery to 4.4 eV. A line profile of this region is shown in the supplementary information (Fig. S5a). The gradual change of the workfunction cannot be attributed to a space charge region (SCR) caused by charged defects at the GB, since lengths on the order of a few micrometers are not likely to occur. The observed change in workfunction can then be attributed to a strain gradient, present in the films in the vicinity of the GBs.

In Fig. 2c, f, a similar situation is shown, where two stripe regions are separated by a GB. The region was especially interesting since here the exact position of the GB was visible even in the topography image. The height changes here were in the range of 5 nm to 10 nm as shown in Fig. S5b. In analogy to Fig. 2b, e, a reduction of the workfunction in the GB vicinity was observed. However, the low work-function region extends over approximately 1 micrometer, and there was also a modification of the surface morphology (see Fig. S4). Once again, charges at the GB are discarded because the workfunction value is constant all over the 1 micrometer region, which is not compatible with SCR. A more reasonable assumption is then attributed to a strain gradient, which changed the workfunction and surface morphology in the vicinity of the GBs. A small gradient in the workfunction all across the 8*μ*m large image could also be measured. The exact reason is not yet clear.

**Fig. 3:**
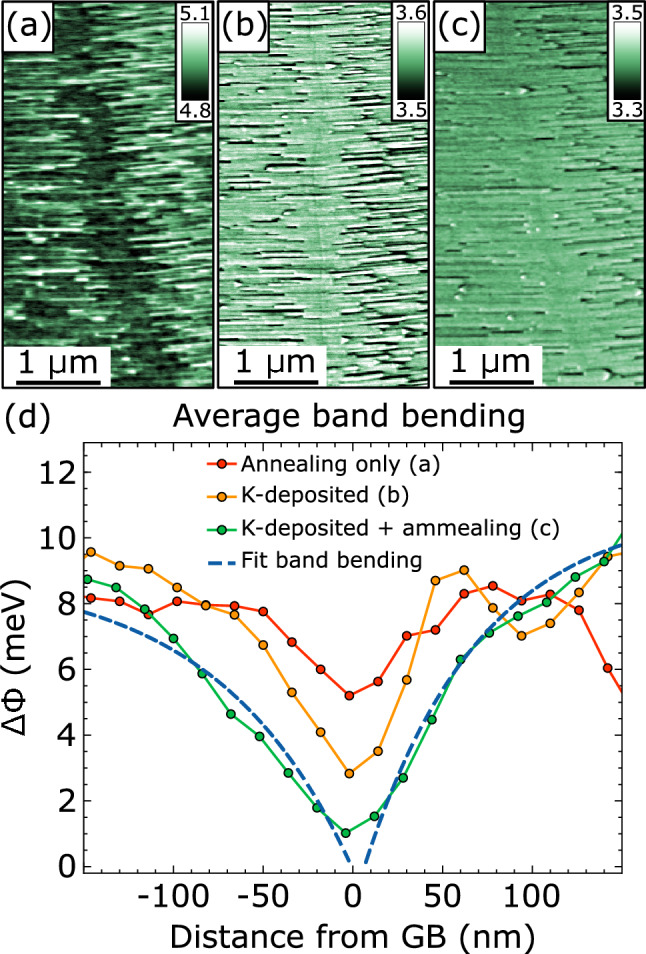
Workfunction maps of the GB depicted in Fig.2 (**c**,** f**) after (**a**) 30 minutes UHV annealing at 200 $\vphantom{0}^\circ$C, (** b**) approximately 3 nm of K deposition, and (** c**) annealing the CIGSe absorber for 60 minutes after K-deposition. (**d**) Average GB workfunction contrast deduced from (**a**), (**b**), and **c**. The dashed line shows a fit to the workfunction variations from (**c**) according to Fig. 1. False color bar units for (**a**), (**b**), and (** c**) in eV.

Although gradual changes in the workfunction were observed in the vicinity of the GBs, no clear contrast of the workfunction confined exclusively to the GB could be measured. As structural changes at GBs, and especially at low-angle GBs, are restricted to a few unit cells^29^, a more prominent and localized contrast was expected. Only in Fig. 2f, a very slight increase in workfunction was found at the GB close to the resolution limit of the KPFM technique (lower part of the image). In Fig. 3, the impact of K deposition and annealing will be discussed in the region shown in Fig. 2c, f.

**Table 1:** Chronological sequence of sample processing steps.

Step	Process stage	Description
1	As-grown	Sample as received from the MOVPE.
2	Annealed	Sample annealed for 30 minutes at $200^\circ$C.
3	K-deposition	3.2 nm of potassium was deposited on the sample surface.
4	K-deposition + anneal	Annealed for 30 minutes at $200^\circ$C.
5	2nd anneal	An additional annealing for 30 minutes at $200^\circ$C.

Figure 3 depicts the KPFM images collected at the same GB as shown in Fig. 2c, f, although after different treatments (see Table 1). As discussed above, the images presented in Fig. 2 were collected directly after growth. X-ray photoelectron spectroscopy (XPS) confirmed that the amount of oxygen adsorbed on the surface was below the detection limit; however, a small amount of residual carbon was detected. After annealing the sample for 30 minutes at 200 $\vphantom{0}^\circ$C the carbon was removed (see Fig. S6) and an increase in the average workfunction of *≈* 420 meV was measured. This change in workfunction can be attributed to the removal of physisorbed carbon-containing adsorbates, which are known to change the workfunction of surfaces due to a change in the surface dipole^30^. A close inspection of the GB region via KPFM showed that the workfunction contrast, observed in Fig. 2f, is not evident after the annealing. In addition, a small increase in the gallium-to-indium ratio at the surface was measured via XPS, which will be discussed later in this manuscript. The results presented here are consistent with a previous report, in which an increase in workfunction after heating in UHV^31^ was measured on CIGSe.

Thereafter, K was deposited on the surface of the CIGSe, which was exempt from any contamination. This procedure was carried out in UHV with the pressure during deposition kept below $5\cdot 10^{-10}$  mbar to ensure a clean metallic K surface. From the Se 3d peak intensities before and after deposition, a thickness of approximately 3.2 nm was deduced (see^31^ for more details on the calculation).

Figure 3b shows the same GB, right after the deposition of K. A strong reduction in the average workfunction was observed compared to the pristine and UHV annealed cases, with an average value of only 3.6 eV. This low average workfunction value was expected, since metallic K has a theoretical workfunction of 2.74 eV^32^. In addition to the reduction of the average workfunction, smoother workfunction variations were also observed. For the clean CIGSe surface, two main components of the workfunction (i.e., the two grains) with full width at half maximum (FWHM) of 60 meV and 130 meV (see Fig. S7) were measured. After K-deposition, these variations were reduced, and the two main workfunction distributions exhibited FWHM of only 33 meV (see Fig. S7). It is expected, considering that the surface is covered with a single metallic element. Interestingly, the exact position of the GB was visible as a fine line in the workfunction map surrounded by the strain-modified region.

K deposition was followed by two annealing steps in UHV for a total duration of 60 minutes at 200 $\vphantom{0}^\circ$C, to promote the diffusion of K into the CIGSe bulk. Two annealing steps were done to check whether a new equilibrium could be reached, i.e. whether the compositions did not change anymore. The results will be discussed in more detail later in the manuscript. Here, only the result of the second annealing step is shown, which can be considered to be close to equilibrium. The workfunction map is shown in Fig. 3c, where a similar result was found, as for the previous K-deposited case (Fig. 3b). The workfunction remained low (3.4 eV), and a further small reduction was observed at the GB.

The low average workfunction after annealing was surprising and different from a previous report, where a recovery to a high workfunction (*≈* 4.5 eV^31^) was found. In the present study, more K was deposited to saturate the bulk and GBs of CIGSe. In^31^ roughly 8Å of K were deposited on the surface of the CIGSe, while in this study the thickness was approximately 32Å.

After K deposition and subsequent annealing, once again a visible workfunction contrast was found at the GB, showing a tiny drop in the workfunction value (see Fig. 3b, c). To increase the signal-to-noise, profile lines for each scan line were extracted (in total 470 lines) and averaged. The procedure for the extraction of the GB workfunction variations is described in detail in the SI (Figs. S8 to S10). The variations in the average potential at the GB are shown in Fig. 3d, where $\Delta \phi$ corresponds to the workfunction differences between the GB and the surroundings.

The red curve in Fig. 3d, depicts the $\Delta \phi$ for the sample that received only the annealing treatment. A small workfunction fluctuation can be noticed in the region of the GB. Such changes fall within the resolution limit of the KPFM, making them invisible in the images; however, the precise methodology used here to extract the line profiles allowed for their visualization. Then, when the K-deposition was done, a more substantial decrease in workfunction was observed at the GB ($\approx 6\,meV$), while a subsequent annealing step strengthened even further the signal to $\approx 9\,meV$. Furthermore, the width of the drop of the workfunction was slightly enlarged for the post-annealed case compared to that for the K-deposited only. There are two possible interpretations of these data. If a metallic K layer was present even after annealing, then the changes in the workfunction at the GBs could only be described by a different number of K atoms at that position compared to the rest of the grain. The shape of the workfunction drop would then be determined largely by the geometry of the tip apex or by a gradient in the density of K atoms in the vicinity of the GB. The second option to interpret the data would be to assume that the metallic K layer reacted with the CIGSe surface and that the near-surface region of the semiconductor was modified. Assuming this scenario, the drop in the workfunction could be interpreted as band bending induced by additional charges present at the GBs compared to the grain surfaces.

In order to compare these measurements with the depth-dependent KPFM measurements shown later, we will assume that the drop in workfunction at the GB was due to band bending. However, a full interpretation of the data will be provided in the conclusion section of this manuscript.

In the case of band bending, the formalism developed by Lee and Mason can be used^33^, who derived analytical expressions that describe band bending in semiconductors. In the present case, downward band bending at the GB compared to the grain surface was assumed. Furthermore, the workfunction of the grains were very low. The situation after K deposition was clear, as low workfunctions for K-covered films were expected. The situation after annealing was less obvious, as the workfunction was further reduced. In any case, the low workfunction was compatible with an n-type semiconductor or a type inverted layer on top of a p-type semiconductor. This did not necessarily mean that the bulk was n-type, but the surface definitely was. Downward band bending in an n-type semiconductor induced by donor centers gives rise to an accumulation SCR. The diffusion potential is then described by^33^:

1 $$\begin{aligned} tanh \left( \frac{e \Delta V_{GB}(x)}{4k_B T} \right) \approx tanh \left( \frac{e \Delta V_{GB}(x=0)}{4k_B T} \right) \cdot exp \left[ -\sqrt{2} \frac{x}{L_{DB}} \right] \end{aligned}$$

The diffusion potential is defined as $\Delta V_{GB}(x)$ with *x* being the distance from the GB. Consequently, $\Delta V_{GB}(x=0)$ is the drop at the position of the GB with respect to the region far from the boundary. The Debye length is defined as $L_{DB}=\sqrt{\varepsilon \varepsilon _0 k_B T/e^2 P_{net}}$, with $k_B$ the Boltzmann constant, *T* the temperature and $P_{net}$ the net excess carrier concentrations at the GBs with respect to the grains. Using $\Delta V_{GB}(x=0)$=9 meV and *\varepsilon*=13.6 the measured curve can be fitted with $P_{net}$ as the only free parameter. Since Eq. 1 only includes differences in the workfunction, the curve will converge to zero for large values of *x*. For the present case, $P_{net}$= 2.3$\cdot 10^{15}$ cm$\vphantom{0}^{-3}$ was deduced for the region on the right side of the grain, and $P_{net}$= 1.7$\cdot 10^{15}$ cm$\vphantom{0}^{-3}$ for the left side of the GB. The average of the two values equals 2.0$\cdot 10^{15}$ cm$\vphantom{0}^{-3}$. We will compare these values to depth-dependent values later in the manuscript. The important message from this part is that the shape of the potential drop can be well described by Eq. 1.

**Fig. 4:**
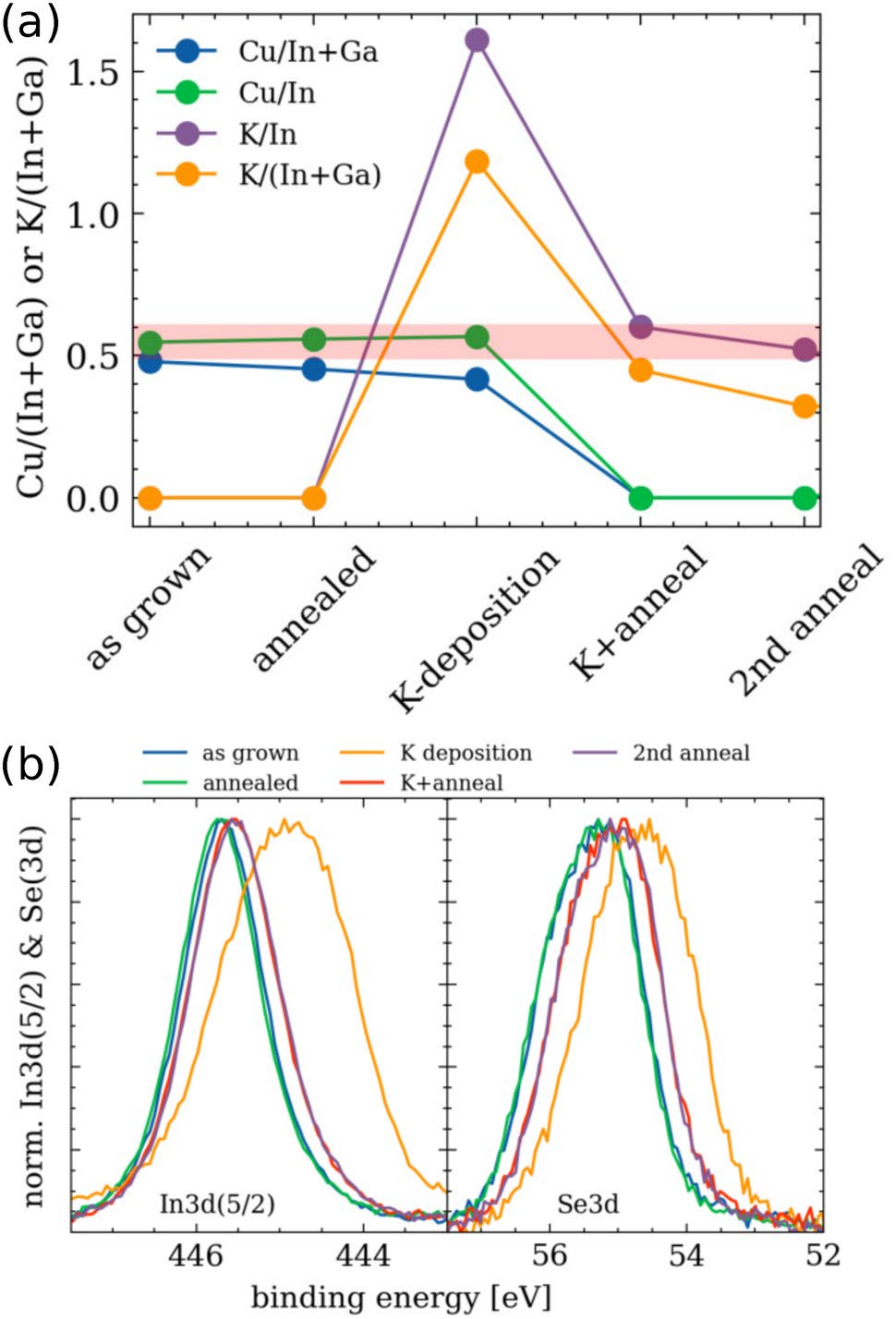
(**a**) Element ratios from XPS after different deposition and heating steps. The shaded area highlights that the Cu/In ratio before K deposition and annealing is equal to the K/In ratio after K deposition and annealing. Each annealing step leads to a slight reduction of the Ga ratio at the surface. (**b**) XPS In3d(5/2) and Se3d core levels for the different heating and deposition steps.

So far, only the results acquired via KPFM have been taken into account. In the next step, the compositional changes induced by the K deposition and the subsequent annealing will be analyzed to better interpret the KPFM results. XPS measurements after each processing step were done on exactly the same samples as those used for the KPFM measurements. Due to the lack of spatial resolution in XPS, the results were averages over multiple grains.

Figure 4a summarizes the variations in the Cu/(In+Ga) ratio, the Cu/In ratio, K/(In+Ga) ratio, and K/In ratio for all annealings and depositions done under UHV. Figure 4b shows high-resolution scans of the In3d(5/2) and Se3d peaks for the different processing steps. The first observation is that the as-grown surface was Cu-depleted in agreement with most other CIGSe reports^34,35^. The reasons for the Cu depletion have been discussed extensively in the literature. The reduction in Cu is usually assigned to the formation of ordered defect compounds (ODC) with a stoichiometry of $\textrm{Cu}_1$ $\textrm{In}_3$ $\textrm{Se}_5$ (denoted as 1-3-5 ODC) or $\textrm{Cu}_1$ $\textrm{In}_8$ $\textrm{Se}_8$ (denoted as 1-5-8 ODC). Another possible explanation discussed in the literature is a completely Cu-depleted monolayer on top of a CIGSe^36^. Both models are compatible with the deduced XPS concentrations, as this technique averages the first 5- 10 nm depending on the element. In the present case, the as-grown samples showed a Cu/(In+Ga) ratio close to 0.5, which can be translated to a 3.2 nm (1,3,5)-ODC layer on top of a CIGSe absorber layer using the same analysis as done by^37^.

After annealing, the Ga at the surface increased slightly, as can be seen from the constant Cu/In ratio and the slight reduction in the Cu/(In+Ga) ratio. Depositing K on the sample surface kept the Cu/In ratio constant, which showed that there was no significant interdiffusion taking place at this step (deposition done at room temperature). The high-resolution peaks of In and Se broadened, with the FWHM increasing by 0.5 eV and 0.4 eV, respectively (Fig. 4b), indicating a reaction of K with these CIGSe elements at the surface. The first annealing after K-deposition led to the complete vanishing of the Cu signal, highlighting that the Cu in the near-surface region was pushed into the bulk. The amount of K at the surface was reduced, indicative of an in-diffusion of K into the bulk. Interestingly, the K/In ratio was already very close to the Cu/In ratio right before the annealing. This is a strong indication that an exchange of Cu with K occurred during the 200$\vphantom{0}^\circ$C annealing. After the second annealing, the ratios did not change much more, which showed that a stable surface composition was reached. The high-resolution scans showed a reduction in FWHM of the peaks after annealing to values comparable to those before annealing.

The XPS results clearly indicate that an exchange mechanism is occurring in the near-surface region, where Cu is replaced by K. The ratios of Cu/In before K-deposition and K/In after K-deposition stayed within a similar range of values, close to 0.5. Furthermore, a gradual increase of the Ga in the near-surface region was observed. The high-resolution XPS peaks corroborated that there was no significant broadening between the peaks of the samples annealed without K and those annealed after K deposition. Consequently, there was no direct indication that a high-bandgap $\textrm{KInSe}_2$ phase was formed on the surface. At least the stoichiometry and width of the peaks pointed toward a Cu-depleted phase on top of CIGSe. However, the XPS averaging effect needed to be taken into account. As already mentioned, the measured XPS ratios are average values over a thickness of approximately 5 nm. Applying the same two-layer model again^37^, this time assuming a $\textrm{KInSe}_2$ layer on top of a $\textrm{KIn}_3$ $\textrm{Se}_5$ yields a $\textrm{KInSe}_2$ of 0.7 nm. This rough estimation showed that a monolayer of a $\textrm{KInSe}_2$ phase could not be excluded.

Independent of a possible monolayer-thick $\textrm{KInSe}_2$ phase on the surface, the Cu-depleted region was much thicker, as the Cu2p signal was below the detection limit after annealing. The minimum thickness can be estimated as follows. The inelastic mean free path of Cu for the core level 2p is equal to 0.7 nm^38^. This distance corresponds to the average distance between two inelastic collisions, and 67% of all Cu2p photoelectrons originate from within 0.7 nm. The 3*σ* threshold (99.7,%) is approximately 2.1 nm, which should be a reasonable estimate of the lower limit of the K-rich, Cu-free layer.

So far, we have assumed that the K diffuses into the CIGSe absorber during annealing. However, this needed to be verified, and therefore, high-resolution helium-ion microscope-based secondary-ion mass spectrometry (HIM-SIMS) was used to study the diffusion of K into the bulk and into the GBs. Measurements were made in multi-successive image acquisition mode. After the acquisition of the first image on the sample surface, successive images were acquired in depth. At the end of the analysis, the total depth was calibrated by an AFM (see SI for details). This depth was then used to calibrate all the other images taken in the intermediate steps. This assumed that the sputtering rate was constant throughout the depth profile. The average sputtering yields calculated with SRIM^39^ for 20 keV Ne$\vphantom{0}^{+}$ ions incident on a $\textrm{CuInSe}_2$ film are 2.4 atoms/ion. For the Cu-depleted phase, the yield increases to 3.0 atoms/ion, and for $\textrm{KIn}_3$ $\textrm{Se}_5$ and $\textrm{KInSe}_2$ it is 2.8 atoms/ion. The calculated sputtering yields vary by at most 25%. Given the complex mix of surface orientations and morphologies, this spread is considered sufficiently small. Such complexity can, in principle, lead to much larger changes in the sputtering yield^40^.

**Fig. 5:**
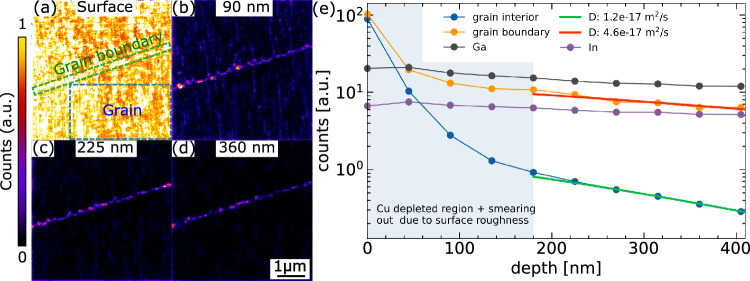
(**a**–**d**) SIMS images (K-signal) as a function of depth for the sample after K deposition and annealing; (** a**) surface, (** b**) after removing 90 nm, (** c**) 225 nm, and (** d**) 360 nm. (** e**) Distribution of K, Ga, and In as a function of depth for the regions denoted as GB and grain interior (**a**). Solid green and red lines show fits to the data with Eq. 2. The sample analyzed here had a thickness in the range of 400-500 nm..

The results of the HIM-SIMS analysis are presented in Fig. 5. The distribution of K on the surface of the CIGSe films is shown in Fig. 5a, while the images acquired at a depth of 90 nm, 225 nm and 360 nm are depicted in Fig. 5b–d. The nanometer-resolved distribution maps corroborate that the near-surface region of the CIGSe film was very rich in K, in accordance with the XPS results. At a nominal depth of 90 nm the K signal was strongly reduced in the grains but was still unevenly distributed, presumably due to angle-dependent sputtering yields. The signal at the GB was much better resolved, as here the amount of K was larger. This trend continued, and at 225 nm and 360 nm only the K at the GB was still visible.

In addition to the variations in K, the Ga and In changes were measured. The changes in the average counts of K, Ga, and In from the grain interiors and from the GBs were analyzed as a function of depth. The results are presented in Fig. 5e on a logarithmic scale. The Ga and In signals were considered as baselines. The profiles were reasonably flat throughout the whole film, and the small changes could be due to slightly different ionization probabilities. The most interesting profiles are those deduced for K within the grain and at the GBs. The profiles were different, which points to different diffusion rates. The two curves could be fitted with the following equation:

2 $$\begin{aligned} C(x,t)=C_0\cdot erfc\left( \frac{x}{2\cdot \sqrt{D\cdot t}} \right) +C_{BG} \end{aligned}$$

which is the solution of the diffusion equation assuming an infinite source of K at the surface of the CIGSe^41^. The coefficient $C_0$ denotes the average concentration of K in the grain or GB, the constant *D* is the diffusion coefficient, and *t* is the annealing time. Profiles beyond 200 nm can be well fitted, given diffusion coefficients of 1.2$\cdot 10^{-17}$ m$\vphantom{0}^{2}/s$ and 4.7$\cdot 10^{-17}$ m$\vphantom{0}^{2}/s$, respectively for grain interiors and GBs, as shown in Fig. 5e. However, the near-surface region is characterized by a non-constant slope. This region was not fitted for the following argument. From the images shown in Fig. 5a–d, inhomogeneities in K on the surface and even after 90 nm were visible. As already outlined, these differences were likely to arise from different sputtering yields due to changes in the local angle of incidence between the ion beam and the surface as a consequence of the facets. Therefore, the changes in the K signal in the first 180 nm were due to different concentrations at different depths. After 180 nm the inhomogeneities decreased and fewer fluctuations in the K signal were observed, which made the extraction of the diffusion coefficient more reliable.

The diffusion coefficient extracted from the GB was approximately three times higher than that of the grain interior. The values are of the same order of magnitude as those of the study by Würtz et al.^41^, who carried out a SIMS analysis on CIGSe absorbers treated with Rb. However, it should be noted that the interpretation and fitting procedure were different. Würtz et al. observed two different slopes in the depth profiles similar to Fig. 5e. They interpreted this to be a convolution of two different diffusion processes, namely GB and intragrain diffusion. The experiments carried out in this work allowed for an unambiguous separation of GBs and grains. Therefore, the two different slopes were not interpreted as GBs and grains since both profiles showed the same trend.

Importantly, the results corroborate that K diffused rapidly into the absorber via the GBs. In addition, an accumulation of K was found in the near-surface region of the CIGSe. The SIMS data also showed that there was diffusion of K into the grain interior, although with a lower diffusion coefficient. The slope of the K signal as a function of depth, shown in Fig. 5e, was less steep than the one at the GB but steeper than the In and Ga profiles. The measurements thus confirmed that the annealing effectively diffused K along the GBs and also in the grain interior of the CIGSe. In the next step, the composition of some GBs was analyzed by APT, and the results were compared to compositional data acquired at the surface of the CIGSe films.

**Fig. 6:**
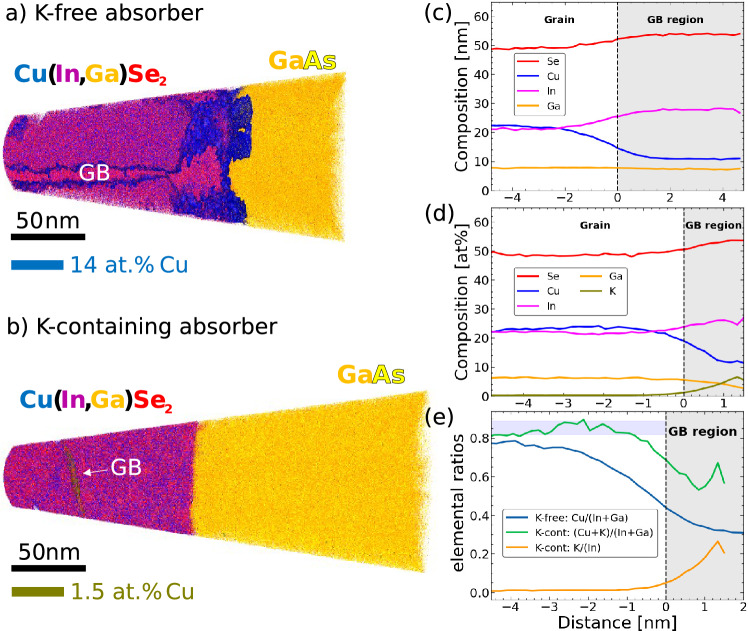
(**a**) 3d APT reconstruction of a GB before K deposition and (**b**) after K deposition and annealing. Corresponding line scans of (**a**) and (**b**) are shown in (**c**, **d**). The GB area and the grains can be separated. (**e**) Comparison of the elemental ratios before and after K deposition.

Figure 6 depicts APT data acquired at a GB in a K-free sample and in a K-containing sample. We were unable to measure the same GB, as preparation of the APT tips was very challenging and we encountered various fractures during preparation and measurements. Consequently, the results can only be discussed on a qualitative level, as there are certainly some variations in GB composition across the film.

Figure 6 a depicts a GB in a K-free absorber normal to the GaAs substrate. The corresponding profile, perpendicular to the GB, is shown in Fig. 6c. The Cu/(In+Ga) ratio is plotted in Fig. 6e in blue. The profiles through the GB could be divided into two regions, namely the GB region and the grain interior region. The interior of the grain exhibits a composition similar to that measured via EDX, namely, Cu/(In+Ga)=0.8. Interestingly, the GB region was very large, almost 10 nm wide. Although the normal orientation of the GB might induce local magnification effects during the field evaporation, this effect does not explain the much wider thickness of the GB solely (See the SI for more details). In fact, the composition was characterized by strong Cu-depletion and In-enrichment behavior, which is close to a (1:3:5) ODC type composition, in agreement with other APT measurements^13^. The composition was also close to the surface composition measured on the same sample via XPS, which was discussed in the previous sections.

In Fig. 6b a 3D APT map of a K-containing GB is presented. The GB was much thinner this time, and although exhibiting the same Cu-depletion and In-enrichment behavior, the composition was strongly deviating from the (1:3:5) ODC compound. In agreement with the SIMS data presented previously, we measured a strong K accumulation at the GB of about 6.5 at.%, which is generally much higher than the one detected in typical CIGS solar cells^16^. The GB showed a reduction in Cu, in agreement with the K-free absorber. In contrast to the near-surface region, the Cu content did not drop to zero right at the GB. The ratios were therefore more complicated to interpret. The K/In ratio increased and reached a value close to 0.2, compared to 0.5 at the surface. The (Cu+K)/(In+Ga) ratio is close to 0.6, whereas at the surface, the value was closer to 0.4. Care should be taken not to over-interpret the differences in ratio, given that XPS exhibits more global composition values, while, on the contrary, APT exhibits more local composition values. Therefore, only trends will be discussed in the following.

The measurements showed that the Cu-content was not zero at the GBs, which appears to be logical considering that the amount of K diffusing from the top into the grain was far inferior compared to the amount evaporated directly on the surface. Consequently, the driving force for Cu to diffuse into the bulk was likely to be lower. The Cu ratio did change, which meant that a redistribution of the Cu into the bulk was likely. Close inspection of the Cu and K profiles in Fig. 6d and of the Cu+K/(In+Ga) ratio showed that the region close to the GB region exhibited a larger amount of Cu compared to the K-free case. In Fig. 6e, the ratio actually surpassed the bulk value slightly at a distance of approximately 2–3 nm away from the GB before it then converged back to the bulk value. It is expected, considering that some of the Cu was pushed into the bulk after the heat treatment. Due to the low-temperature annealing ($200^\circ$C), the diffusion of Cu into the bulk was not high enough to evenly distribute the material in the bulk, which led to a maximum in the ratio in proximity of the GB. Considering that PDTs during device processing are performed at higher temperatures ($350^\circ$C), it is very likely that under such conditions the Cu variations at the GBs are smeared out in the absorbers.

The important implication of this result was that the low-angle GBs studied in this work are only partially decorated with K, and that a direct indication was found that the mechanism of Cu redistribution due to K was identical at the surface and at the GBs. The magnitude of the Cu redistribution probably depends on the amount of K available.

**Fig. 7:**
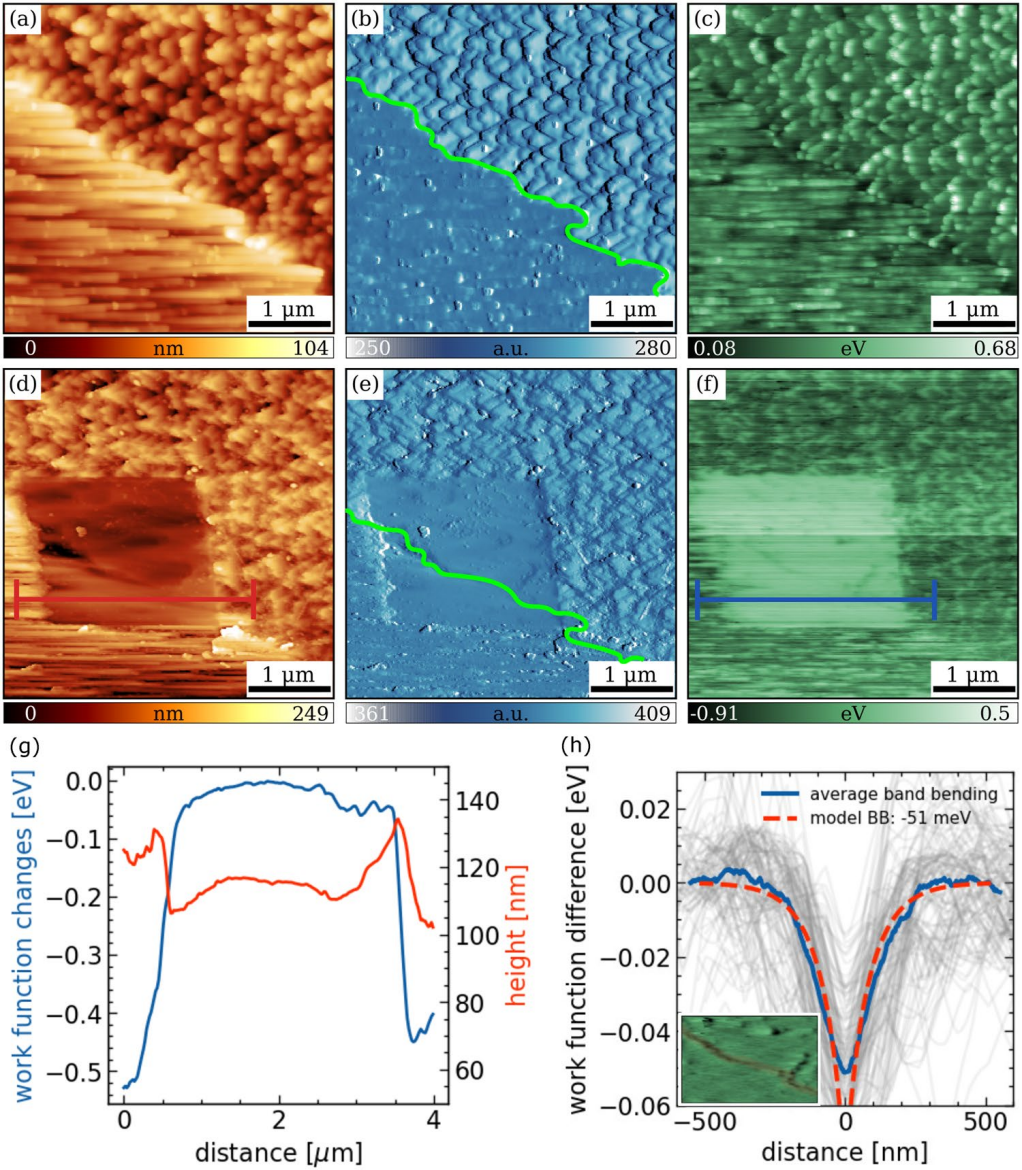
(**a**) AFM topography, (**b**) Amplitude error signal, (**c**) workfunction variation map of a CIGSe region separating a stripe-like region from a pyramid-like region. (**d**–**f**) same region and measurement signals after removing 30 nm of CIGSe with a diamond tip. The profile lines of the topography and KPFM are shown in (g). (h) Variations of the workfunction at the position of the GB, gray: individual profile lines, blue: average profile line, red dashed line: fit to the data according to Eq. 1. The sample analyzed here had a thickness in the range of 400-500 nm.

An important follow-up question was how the surface-sensitive measurements performed with AFM and KPFM can be compared with those of the APT and SIMS, since here, GBs in the bulk were studied. Is the band bending observed on the surface of the CIGSe equally small in the bulk, or is the residual K, the K containing ODC, or even a monolayer of $\textrm{KInSe}_2$ on the surface masking the band bending? In order to answer this question, the AFM tip was used to remove the upper part of the absorber surface to measure a GB deeper in the semiconductor. For it, a tip with a diamond apex was used that did not wear off when scanned in contact mode. Although not calibrated, the applied force was kept constant at less than 1 *μ*N such that 15 nm of material was removed per scan. This is similar to the 14 nm observed by Sharma et al., who found that this did not promote a phase transition in the CIGSe^42^.

The results of the measurements are shown in Fig. 7. A GB was analyzed that separated a stripe-like region and a pyramid-like region, as shown in Fig. 7a. To accurately identify the position of the GB, the topography feedback error signal was used, as shown in Fig. 7b. The green line highlights the boundary between the two regions. The KPFM measurements are shown in Fig. 7c. The measurements are presented in the same way as before (bias applied to sample), which means that higher values of the contact potential difference indicate a higher workfunction. The measurements were not calibrated on HOPG this time, so that only qualitative changes in workfunction can be discussed, not the absolute value. In agreement with the previous measurements, no clear signal was observed at the GB on the surface of the CIGSe.

After measuring the pristine region, the cantilever with the diamond tip apex was used to remove approximately 30 nm of the material. The result is shown in Fig. 7d–f. Profile lines were drawn parallel to the fast scan direction from the topography and KPFM images (Fig. 7d, f) and are shown in Fig. 7g. Despite the removal of only 30 nm, a large increase in the relative workfunction of approximately 500 meV (blue line) was observed. This increase showed that the surface, which was covered with some K and/or Cu-depleted K-enriched material, led to a low workfunction, as already discussed in the previous paragraphs. The data shown here corroborate the validity of this assumption, and the electronic properties inside the CIGSe were different.

In addition to the large change in the average workfunction, there was also a small contrast in the position of the GB. A zoom-in of the scratched region is shown in Fig. 7h. Here, the contrast was maximized and a line-to-line noise reduction algorithm was used to enhance the contrast^43^.

Next, the GB potential drop all along the red line, shown in the inset, was extracted perpendicular to the GB in each pixel along the GB. For each line profile, the minimum value was identified, and approximately 100 curves are shown in light gray in Fig. 7h. Out of 158 initial line profiles, 121 were retained for final analysis. Profiles were filtered based on a 200 nm spatial tolerance relative to the GB location, verified via visual KPFM inspection to correct for automated detection artifacts. The whole data treatment procedure is described in Fig. S11. Although there is quite some scattering between individual line scans, an apparent drop in workfunction could be identified at the position of the GB. The average of all curves is shown in blue, and a downward band bending value of 51 meV was measured. The outlier removal procedure had a minimal impact, shifting the calculated band bending from 51 meV to 49 meV. A fit to the average band bending curve with Eq. 1 allowed to estimate the net excess charge carrier density ($P_{\textrm{net}}$= $1\cdot 10^{15}$ $cm^{-3}$) and the Debye length ($L_\textrm{DB}$= 125 nm). These values can then be used to calculate the number of charged defects at the GB via^33^.

3 $$\begin{aligned} N_D=\frac{\sqrt{2} \cdot P_{\textrm{net}} \cdot L_\textrm{DB} \cdot e \cdot \Delta V_{GB}}{k_B T} \end{aligned}$$

Introducing V$\vphantom{0}_\textrm{GB}$=51 meV into Eq. 3 yields a value of N$\vphantom{0}_\textrm{D}$=3.5*·* 10$\vphantom{0}^{10} \pm$ 1.2*·* 10$\vphantom{0}^{10}$ charges/$cm^{2}$. The error bar was deduced as follows. A constant net doping density of $P_{\textrm{net}}$= $1\cdot 10^{15}$ $cm^{-3}$ was assumed and for each profile shown in Fig. 7h, the variation in CPD was deduced. Using Eq. 3, the density of charges could be calculated, and the standard deviation of that value was then used for the error bar of the mean value given above.

Due to the complexity of the measurements, we do not have sufficient statistics to claim that the value of the charge densities is general for all GBs present in this study. Therefore, the results are only discussed qualitatively. The measurements showed that any residual K or K-enriched phase strongly reduced the workfunction of the CIGSe. This reduction, which is a consequence of a reduced surface dipole or a different phase, led to low values of the GB potentials. Removing the top surface drastically increased the workfunction, and larger changes in the workfunction at the GBs could be measured. Importantly, there was no sign reversal, i.e., downward bending on the surface, and also 30 nm below the surface was found. We therefore concluded that the measurements carried out on the surface did give valuable information about the band bending direction, even though the absolute values of the GBs potentials were too low.

**Fig. 8:**
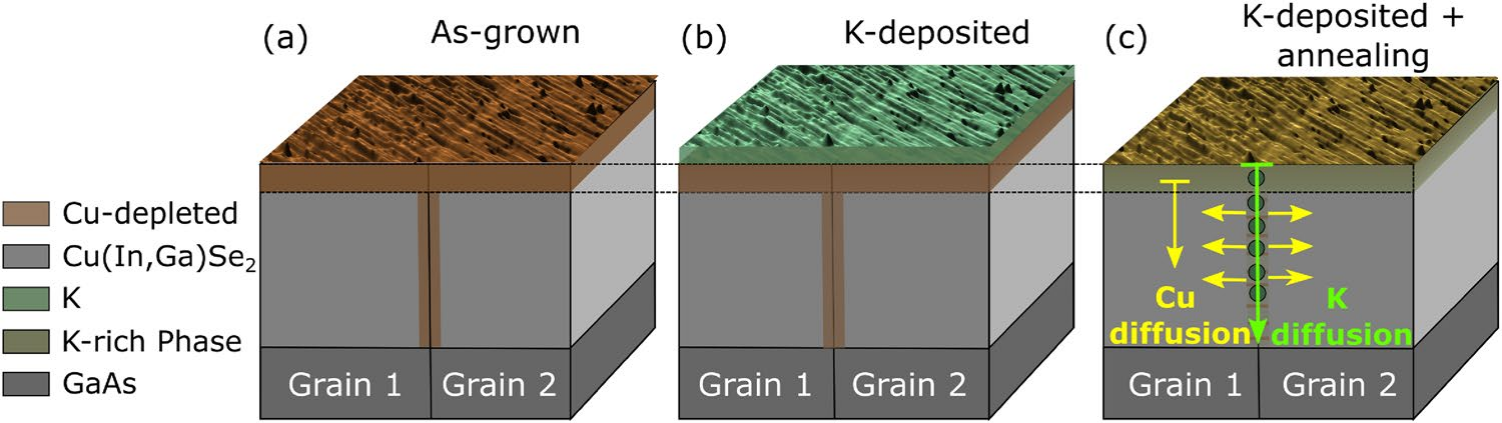
Sketch of the surface and GB before and after K deposition and annealing. The arrows indicate which elements diffuse at which step. For details, see the text.

The results obtained from the present study are summarized in Fig. 8. The surface of the as-grown CIGSe exhibited a pronounced Cu depletion, which is in complete agreement with most of the CIGSe literature. in Fig. 8a the Cu depletion is illustrated in a brownish color, while the bulk, with a Cu/(In+Ga)=0.85 composition, is shown in gray. The GBs were not exempt from a pronounced Cu-depletion, as shown by compositional profiles measured via APT. We found that the compositional values at the surface and at the GBs were very close to each other, suggesting that the same phase/segregation formed at both interface types.

After K deposition at room temperature, the composition of CIGSe in the near-surface region did not change. We observed a strong decrease in the work function, due to a change in the surface dipole, which is expected for K. K formed a homogeneous layer on top of CIGSe, as illustrated by the green color in Fig. 8b.

After annealing, we observed an in-diffusion of K into the GBs and, to a lesser extent, also in the grains. The surface properties of the CIGSe changed substantially, and Cu could no longer be detected after the heat treatment in the near-surface region, as corroborated by XPS. Since the possibility of Cu evaporation from the surface at $200^\circ$C could be ruled out, we deduced that Cu was pushed into the bulk of the CIGSe. This is illustrated with a yellow arrow in Fig. 8c. Since we detected a clear diffusion profile of K in the bulk of the CIGSe we could also assume that some amount of K diffused from the near-surface region into the bulk (smaller white arrow in Fig. 8c).

At GBs, the diffusion of K was faster and extended all the way to the bottom of the films, which were in the present case approximately 500 nm thick. This was corroborated by SIMS depth profiles and indicated with a larger white arrow in Fig. 8c. For both cases (bulk and GBs) we did not see a flat K profile, which means that more K was present in the near-surface region than in the bulk. This conclusion cannot be generalized to all CIGSe films subjected to PDT, since here we restricted the annealing temperature to $200^\circ$C.

In addition to the diffusion of Cu from the surface into the bulk, we also found a substantial residual K concentration after annealing. We measured a K/(In+Ga) ratio that was very close to the Cu/(In+Ga) ratio that we found prior to the annealing. This is a strong indication that Cu is exchanged by K. A result that was also already reported for polycrystalline films^6,21^. The measurements therefore suggested that the Cu depleted phase was modified and consisted mainly of K,In and Se. A thick $\textrm{KInSe}_2$ layer could be ruled out, based on the XPS compositions, but a monolayer of $\textrm{KInSe}_2$ could not be excluded due to the averaging effect of the XPS.

At the GB, we found a substantial accumulation of K. Profiles of the Cu-ratio measured from the GB into the bulk exhibited a maximum near the GB, which was not observed for the K-free case. In analogy to observations on the surface, we propose that Cu at the GBs was pushed into the grains and partially exchanged by K (indicated by smaller yellow arrows in Fig. 8c). However, the exchange was incomplete, and therefore, the Cu content did not reach zero at the exact position of the GB. A possible explanation could be that there was not enough K available to fully exchange with Cu in the GB, in contrast to the situation in the near-surface region.

The proposed mechanism had implications for the GB band bending that was measured with KPFM at the surface of the CIGSe. Both the surface and the GBs change in a similar manner when exposed to K. Consequently, the KPFM measurements did not reflect a band bending between the CIGSe (bulk) and the GB, but rather a band bending between a Cu-depleted/K-enriched phase that is found at the grain surface and the same phase at the GBs. This is also reflected in the very low values of GB band bending that was measured. We could only detect an extremely small value of the order of 10 meV, which is close to the detection limit of the technique.

Most importantly, this band bending is not characteristic of band bending between the CIGSe interior and the GB. The surface and the GB at the surface are, in fact, very similar in composition and also in electronic properties. The small amount of downward band bending is presumably a consequence of a slightly higher K concentration at the GB compared to that at the surface.

The depth-dependent measurements after the removal of 30 nm corroborated this assumption. We find higher work functions of the CIGSe and the amount of band bending increased. Although we did not have enough statistics to generalize the findings to all GBs, we consider these results as extremely important, since they show that ODC and residual K can change measured band bending values significantly at the surface which cannot be translated to band bending in the bulk of the films.

Our measurements and analysis suggest that we do have diffusion of K and Cu into the bulk of the CIGSe. Both elements will have an impact on defects in the CIGSe absorber layer. According to the literature, p-doping is driven by Cu vacancies and Cu$\vphantom{0}_{III}$ anti-side defects and the compensating donors are probably Cu interstitials and In$\vphantom{0}_Cu$ anti-side defects, which are shallow donors^44^. It is also known from literature that the compensation is lower if the Cu depletion is reduced^45^. Furthermore, it was recently shown that KF treatments performed on epitaxial CIGSe increase p-doping, which can be promoted by an increase in acceptor concentration or a decrease in donor concentration^46^. A peculiarity of the epitaxial CIGSe samples is that they are often n-type because of Se depletion, which occurs inherently in the MOVPE process because of the lack of suitable Selenium precursor reactivity during cool-down^47^. The Cu driven into the bulk could obviously reduce the Cu vacancies. However, the changes will be rather small. If we assume that all Cu atoms in the near-surface region (3*×* IMFP of the Cu(2p)=2 nm) are driven into the bulk, then the composition would only change by 0.1%, assuming a CIGSe chalcopyrite crystal structure, a (1,3,5) composition and a thickness of the (1,1,2) phase of 500 nm. This number is low and cannot be resolved with an averaging technique such as energy-dispersive X-ray analysis. Furthermore, a reduction in the number of Cu vacancies would reduce p-doping. Cu interstitials are also not likely since this would increase the donor concentration and consequently reduce the net p-doping. As a consequence, Cu migration in the bulk, which was observed in the present study, is probably not the cause of an increase in net doping, as observed in KF-treated epitaxial CIGSe absorbers. K-induced changes in doping are therefore expected to be dominant. However, Cu vacancies play an important role in the diffusion of K into the bulk and also into the GBs.

## Summary

In summary, we showed how epitaxially grown CIGSe on multicrystalline GaAs can be used to study GBs in a very effective way. Individual GBs extending over hundreds of micrometers formed, which allowed for an accurate statistical analysis. We used this model system to study the interaction of K with a multicrystalline CIGSe surface in detail and could derive a detailed model of the competing processes that occur during such a PDT treatment. The results show how a phase-segregated surface and GB region obscured the true GB band bending. AFM tomography measurements prove to be versatile for studying GB potentials deep in the bulk. From our proof-of-concept study, we see that the band bending values are likely to be larger in the bulk than at the surface. Furthermore, compositional measurements carried out by XPS at the CIGSe surface and APT measurements from GBs showed very similar values, which strongly suggest that the segregation and the interaction of K with the CIGSe are similar at the surface and the GBs. Nanometer-resolved SIMS measurements allowed us to deduce the different diffusion coefficients for the GB and the CIGSe bulk.

The model system used here should be extended to other alkalis, and especially the interaction of Na and K/Rb could be very interesting, since here an ion-exchange mechanism was suggested in the literature^13^. AFM, KPFM, and HIM-SIMS could be excellent tools to follow this exchange. Laterally resolved diffusion coefficients could be measured in a way similar to the methodology used in this work, and this knowledge would certainly be very important for further device optimization. Finally, the structural and electronic variations we studied in this work need to be supplemented with optical measurements to quantify how band bending translates into non-radiative losses. In the present case, this was not possible since the CIGSe PL emission was much weaker than that of the underlying GaAs. Thicker samples would be necessary to carry out such an analysis.

## Methods

Epitaxial Cu(In,Ga)$\textrm{Se}_2$ samples were grown using metal-organic vapor phase epitaxy (MOVPE). The growth process was adapted from^48^, with a third step introduced at the end of the growth to finely tune the copper and gallium contents. Pieces of epi-ready Zn-doped GaAs(001) from Wafer Technology LTD and microcrystalline GaAs substrates from CMK s.r.o. were simultaneously introduced into the MOVPE reactor. Unless otherwise specified, the sample thickness was approximately 200–300 nm after a growth process of 4.5 hours. Thicker samples (same growth parameters, twice the time), with a thickness of 400–500 nm were analyzed using the HIM-SIMS and AFM tomography to better understand the diffusion process of K along the GBs.

All the analyzed samples were Cu-poor, with *Cu/(In + Ga)* ranging from 0.85 to 0.97, and *Ga/(Ga + In)* ranging from 0.30 to 0.34. After growth, samples were transferred from the N$\vphantom{0}_2$-filled glovebox connected to the MOVPE to a second glovebox by using a tight N2-filled container. The sample was then cleaved and transferred to an UHV system. Thus, exposure to ambient air was avoided, and oxidation was kept to a minimum. A detailed discussion will be given in the XPS section of this manuscript.

Metallic evaporation of K in UHV was performed with an alkali metal dispenser mounted into a home-built evaporator. Details of the evaporation procedure can be found in^31^.

Atomic force microscopy measurements (AFM) and Kelvin probe force microscopy measurements (KPFM) were carried out in a variable temperature UHV system (Omicron) with a base pressure of 1-2*·* 10$\vphantom{0}^{-10}$ mbar. Non-contact AFM was used to measure the topography and cantilevers (Si cantilevers with a Pt/Ir coating, 240AC-PP - OPUS) with a nominal resonance frequency of 70 kHz were used for most measurements. Measurements were carried out at a frequency shift of -3 to -5 Hz, while keeping the amplitude of the cantilever resonance constant via a phase-locked loop. To acquire more trustworthy workfunction data and to avoid morphology crosstalk, the KPFM measurements were performed in frequency modulation mode (FM-KPFM) and acquired in a single pass with the topography^27,49^. An AC-voltage of 0.4 V was used for side-band excitation approximately 1 kHz away from the resonance frequency. The lock-in sensitivity was set to 10 mV with a time constant of 1 ms. To measure the surface workfunction, a DC voltage was applied to the sample to nullify the electrostatic force gradient between the tip and the sample. The tip workfunction was calibrated on highly oriented pyrolytic graphite, stored in UHV, and cleaved in regular intervals. The color scales of the workfunction maps are such that brighter regions correspond to larger workfunctions.

AFM tomography was carried out in N$\vphantom{0}_2$ environment using a NanoScope V (Digital Instruments) with highly doped diamond cantilevers (AD-2.8-AS - Adama Innovations). With the AFM in contact mode and gradually increasing the applied force, approximately 30 nm of material was removed from the top surface. Although KPFM measurements with the same tip were possible, we observed a reduction in image quality after removing the CIGSe top layer. Therefore, the tip was exchanged, and the same scratched region was measured. For these measurements, the tip workfunction was not measured and, consequently, the presented data show changes in workfunction instead of absolute values. The color code is adjusted such that brighter regions represent higher workfunction values.

Large-scale AFM topography images, as shown in Fig. 1 were carried out in N$\vphantom{0}_2$ environment in intermittent contact mode using a NanoScope V (Digital Instruments).

X-Ray photoelectron spectroscopy (XPS) was performed in the same UHV system as the AFM/KPFM measurements. A non-monochromatic MgK*α* X-ray source was used for the measurements, operated at a voltage of 12 kV and an X-ray emission current of 30 mA. On the detector side, a pass energy of 50 eV was used with a slit of 0.5 *×* 25 mm. The spectra shown in this work were acquired with a step energy of 0.1 eV. A detector transmission function was deduced to guarantee that the reported surface compositions were accurate. The following samples were used to cover the relevant kinetic energy range: Au(001), Ag(001), Cu-foil, GaAs. The metals were sputtered in UHV, followed by annealing, whereas the GaAs was heated in UHV until the oxygen contamination was removed. All intensities were corrected with the tabulated elemental sensitivity factors^50^ and the inelastic mean free path^51^ for the respective element and kinetic energy. CASA XPS^52^ was used to analyze the data. The background was fitted with a spline Shirley and the sum form of the Gaussian/Lorentzian (SGL(40)) line shape for all elements^53^.

High-resolution (secondary ion mass spectrometry) SIMS measurements were performed with a ZEISS NanoFab Helium ion microscope (HIM) equipped with a magnetic sector system^54^. A 20 keV-focused Ne beam sputtered the surface and simultaneously acquired SIMS images. The lateral distribution of K, In, Ga, and Se was measured at different depths of the absorber layer. The lateral resolution was approximately 20 nm^54^. At the end of the analysis, the sample was transferred to an AFM instrument, which was used to calibrate the depth of the crater. Thus, the elemental distribution could be analyzed as a function of depth.

The EBSD measurements were performed with the EDAX detector using a DigiView 5 camera. Since the sample surface was very smooth, no sample preparation was required. The samples for the APT measurements needed to be prepared as a very thin needles with a diameter of less than 100 nm at the apex. The preparation method for thin needles was the “lift-out method”^55^. The CIGS lamella was obtained by employing an accelerating voltage of 16 kV and then placed on a Si coupon by Pt deposition in the FIB. The radius of the APT sample was reduced by employing the “annular milling” procedure until the sample reached a diameter < 60 nm. Finally, the sample was cleaned under low-beam conditions (e.g. 5 kV acceleration voltage and 23 pA current) to minimize the contamination and amorphization with Ga$\vphantom{0}^+$. Moreover, the APT measurements were performed at temperatures between 20 K and 50 K (cryogenic temperature), to avoid the migration of atoms to the surface of the needle-shaped specimen. The UV laser pulses with a wavelength of 355 nm and a laser energy of 3 pJ (250 kHz laser frequency) were applied to the tip.

## Supplementary Information

Below is the link to the electronic supplementary material.

Supplementary Information.

## Data Availability

The data that support the findings of this study are available from the corresponding author upon reasonable request.
