# Supplementary material for: The influence of potassium post-deposition treatments on grain boundaries and surfaces of Cu(In,Ga)Se2 absorbers
Source: Sci Rep. 2026 Jun 3;16:25265. doi: 10.1038/s41598-026-55190-9 (PMC13469111; doi:10.1038/s41598-026-55190-9)
Supplement: Supplementary file 1 [file 41598_2026_55190_MOESM1_ESM.pdf]

The Influence of Potassium Post-Deposition Treatments on Grain Boundaries and Surfaces of $Cu(In,Ga)Se_2$ Absorbers

Evandro Martin Lanzoni[1], Omar Ramírez[1], Muhammad Uzair Farooq[1], Oana Cojocaru-Mirédin[2], Jean-Nicolas Audinot[3], Tom Wirtz[3], Susanne Siebentritt[1], and Alex Redinger[1,*]

[1]Department of Physics and Materials Science, University of Luxembourg, Luxembourg, Luxembourg

[2]Albert-Ludwigs Universität, INATECH, Freiburg, 79110, Germany

[3]Advanced Instrumentation for Nano-Analytics (AINA), Luxembourg Institute of Science and Technology (LIST), Esch-sur-Alzette, L-4362, Luxembourg

[*]alex.redinger@uni.lu

ABSTRACT

Alkali metal post-deposition treatments (PDTs) have played a pivotal role in the development of high-efficiency $Cu(In,Ga)Se_2$ thin film solar cells. The PDTs impact the near-surface region of the absorbers, the grain boundaries, and the bulk. Since $Cu(In,Ga)Se_2$ solar cells are polycrystalline, the investigations turn out to be very challenging as each grain/grain boundary is affected slightly differently by the PDTs. Therefore, we synthesized $Cu(In,Ga)Se_2$ absorbers on microcrystalline GaAs substrates to form millimeter-sized epitaxial domains with well-defined grain boundaries. This model system allows us to study the same surface orientation and grain boundary before and after PDTs. We find strong preferential (112) facets and Cu depletion on the surface, with the grain boundaries exhibiting very similar Cu-depleted compositions. After K deposition and annealing, Cu is pushed into the absorber, leaving a K-rich compound at the near-surface. Furthermore, we find different diffusion rates of K through the grain interior and via the grain boundaries.

The exchange of Cu with K at the surface also occurs at the grain boundaries as measured with atom probe tomography. High-resolution KPFM measurements performed on the same grain boundary before and after PDT treatment show almost negligible grain boundary band bending. Cu-depleted K-rich area at the surface and grain boundaries exhibit very similar compositions, which gives rise to small band bending values. Our results give an unprecedented view of the mechanisms that occur at the surface, grain boundaries, and the bulk of CIGSe solar cells, paving the way to further develop the materials and improve the power conversion efficiency.

Supplementary Information

The supplementary information contains the following supporting topics:

S1. Electron Backscatter Diffraction quality maps.

S2. Region of the wafer where the KPFM measurements were done.

S3. Zoom-in of the Topography and KPFM at the GB from Figure 2(b,e) of the main manuscript.

S4. Morphology and workfunction changes near the GB.

S5. X-ray photoelectron spectroscopy measurements showing the removal of the carbon contamination after the annealing procedure.

S6. Workfunction distributions during K deposition steps.

S7. Procedure for the extraction of the GB workfunction variations from the KPFM images.

S8. Evaluation procedure: depth-dependent KPFM measurements.

S9. SIMS measurements; additional information.

S10. APT measurements; additional information.

S1 Electron Backscatter Diffraction quality maps

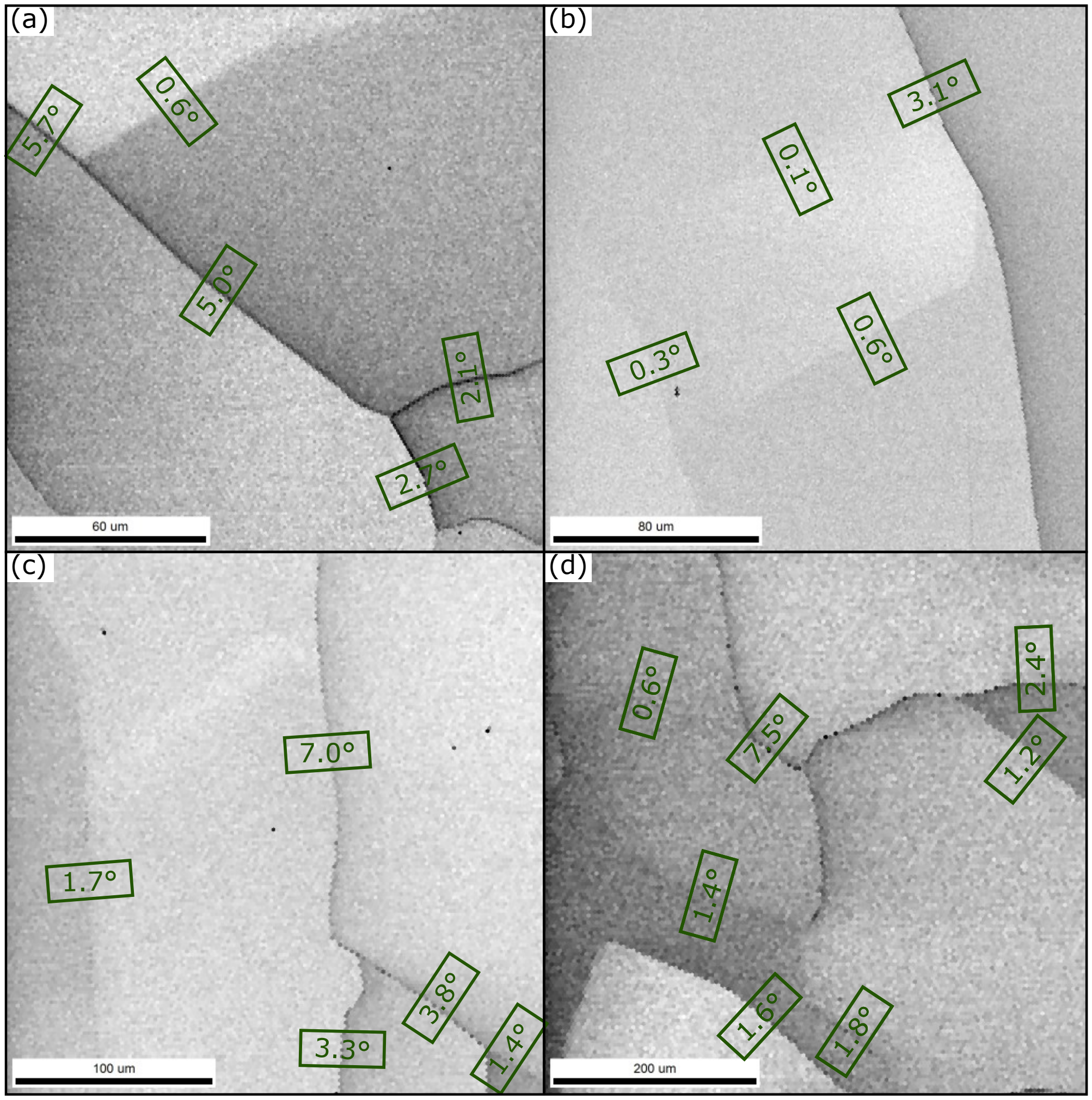

Figure S1. Electron Backscatter Diffraction (EBSD) image quality maps acquired on 4 different regions of the GaAs multicrysralline substrate.

Electron Backscatter Diffraction (EBSD) image quality maps were acquired on different regions of the GaAs multicrystalline substrate. Small deviations from the main orientation $(111)_{cub}$ were observed, showing that the substrate is mainly composed of low-angle GBs.

S2 Region of the wafer where the KPFM measurements were done

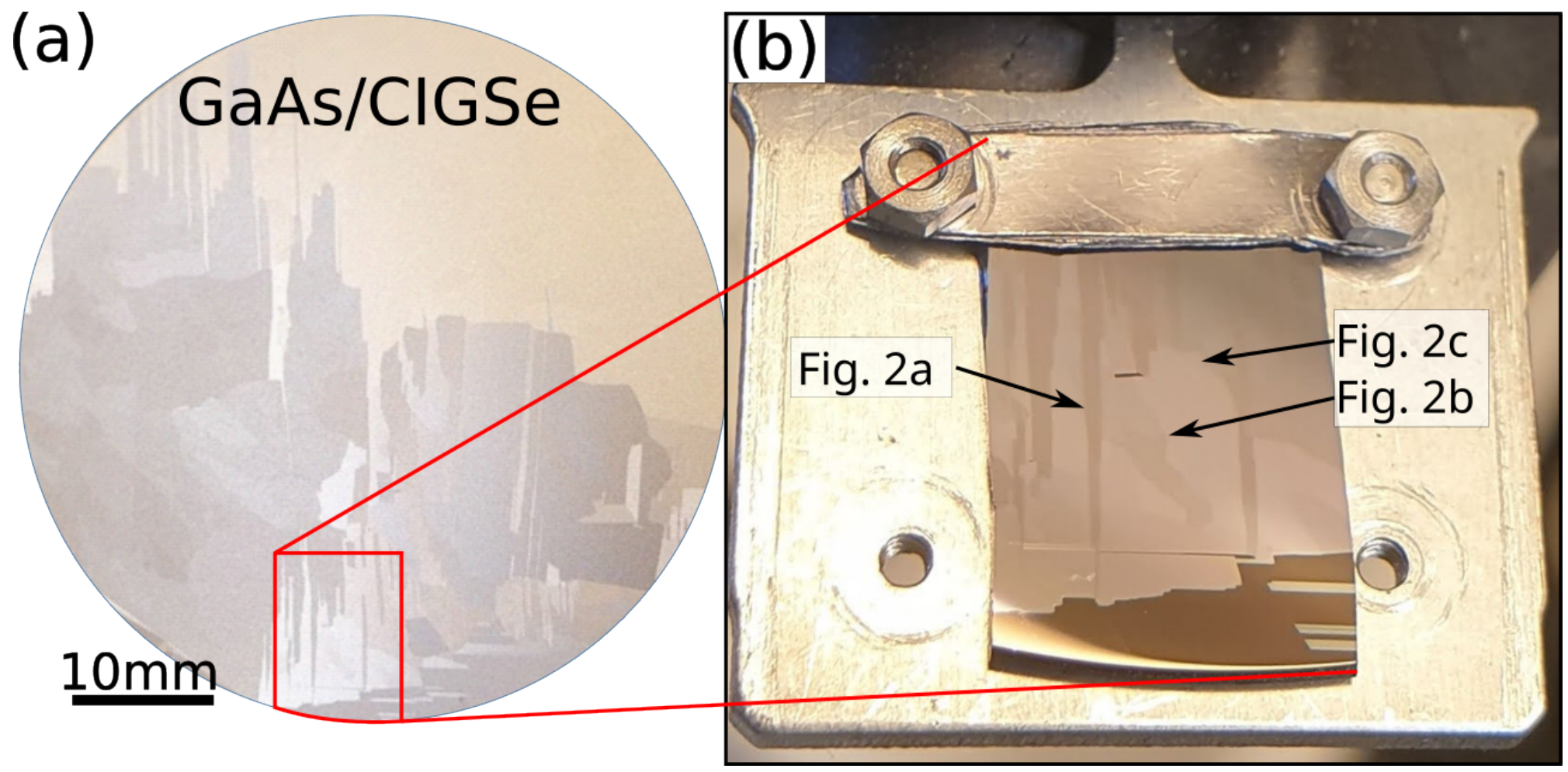

Figure S2. Photograph of the GaAs wafer that was used for the analysis of the KPFM. The analyzed GBs shown in the main manuscript are highlighted.

Figure S2(a) shows a photograph of the wafer that was used for the KPFM measurements. The wafer had to be cut in order to fit in the UHV KPFM system. This was carried out in the glovebox. Afterwards, the sample was mounted on a flag-style sample holder (Figure S2(b)), which was then transferred to the UHV system with the help of an inert gas transfer system. The highlighted GBs in Figure S2(b) correspond to the ones shown in the main manuscript Figure 2.

S3 Zoom-in of the Topography and KPFM at the grain boundary.

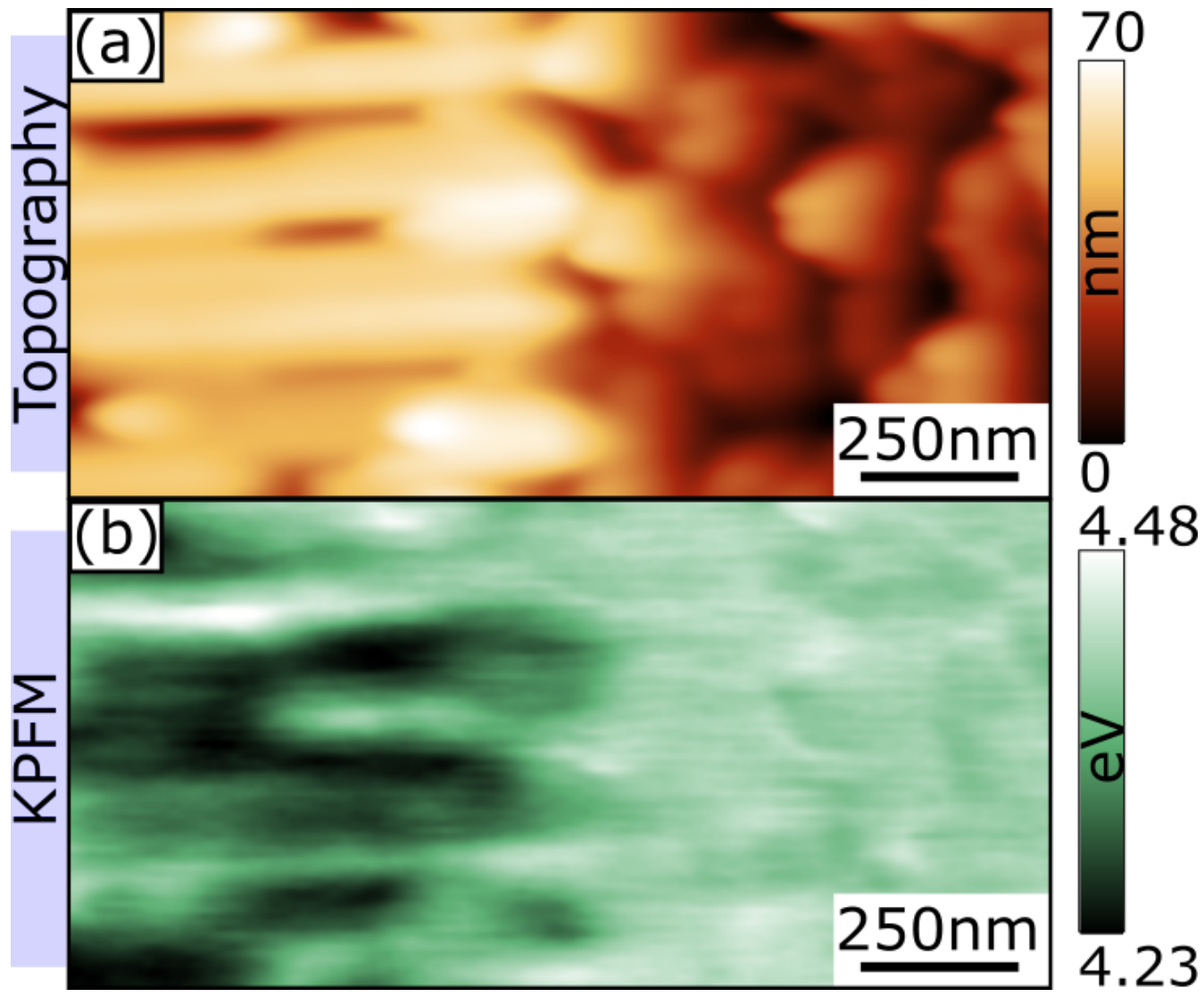

Figure S3. Zoom-in of the (a) Topography and (b) KPFM extract from Figure 2(b,e) of the main manuscript.

Figure S3 shows the zoom-in topography (a) with the respective KPFM image (b) in the surrounding of the GB. The zoomed-in images provide better evidence of the similarity in the workfunction values between some regions of the stripe-like pattern and the pyramid-like structures, which are assumed here to be the $(112)_{tet}$ orientation of the CIGS.

S4 Morphology and workfunction changes near the grain boundary.

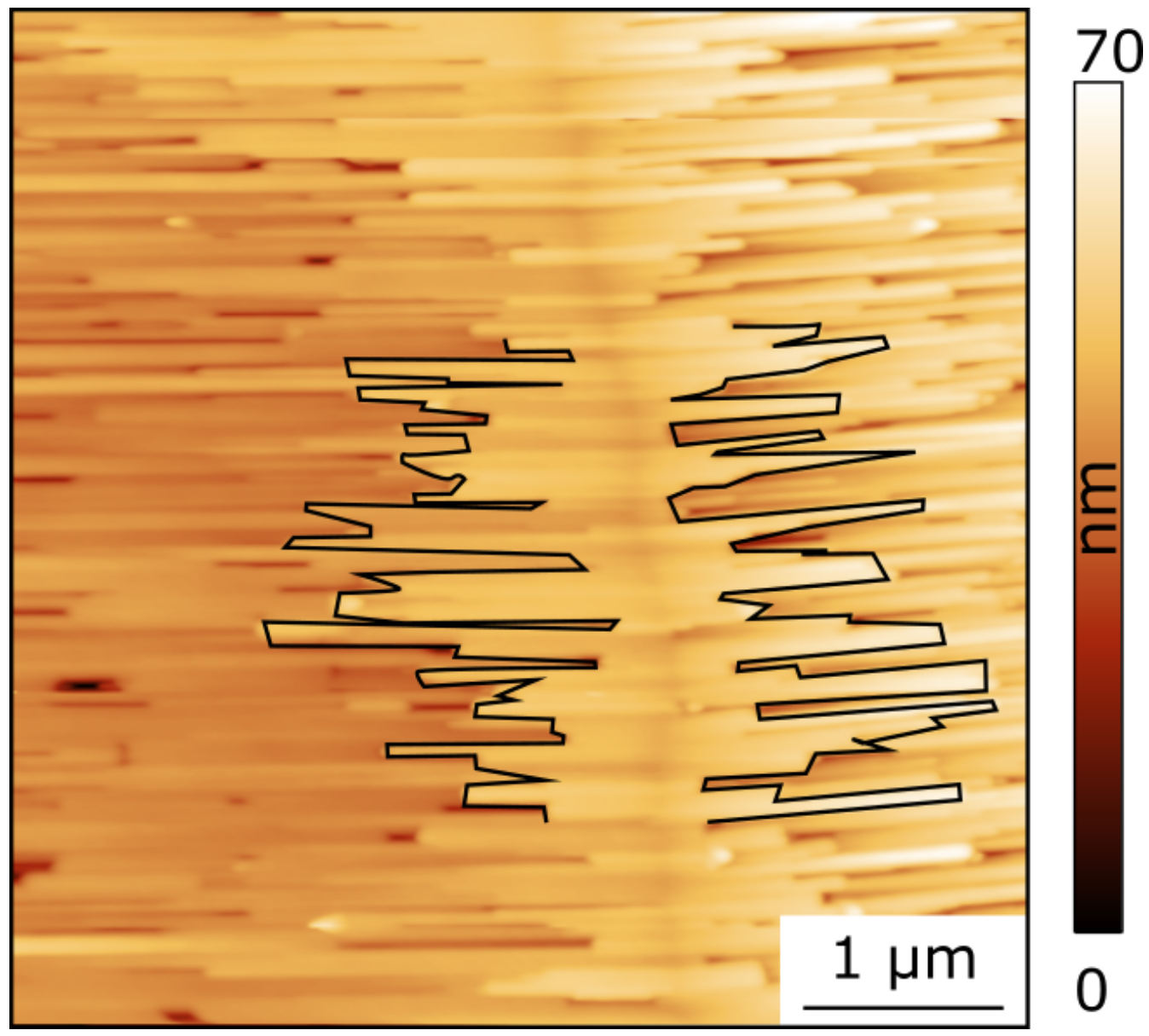

Figure S4. Topography showing a distinct morphology in the surroundings of the grain boundary. The depicted image is a zoom-in of Figure 2 (c) from the main manuscript.

Changes in morphology near the GBs were observed for almost all measured GBs in this manuscript, some extending only a few nanometers and others up to 8 μm. Strain is assumed to be responsible for altering the sample morphology and consequently the surface potential. An example of these distinct morphologies is depicted in Figure S4, which is a zoom-in of Figure 2(c) discussed in the main manuscript. A dark line was used to highlight the contours of the distinct area.

Figure S5 depicts line profiles from the topography (blue) and workfunction (orange) maps and extracted perpendicular to the GBs. Figure S5 (a) refers to line profiles extracted from the images in Figure 2 (b,e), and it shows a gradual change in workfunction over 8 μm starting from the grain boundary. While Figure S5 (b) from the images in Figure 2(c,f), and a sharp change in workfunction over an area within 1 μm from the grain boundary. Also interesting to note here is the very small height step, of less than 5 nm, in the grain boundary.

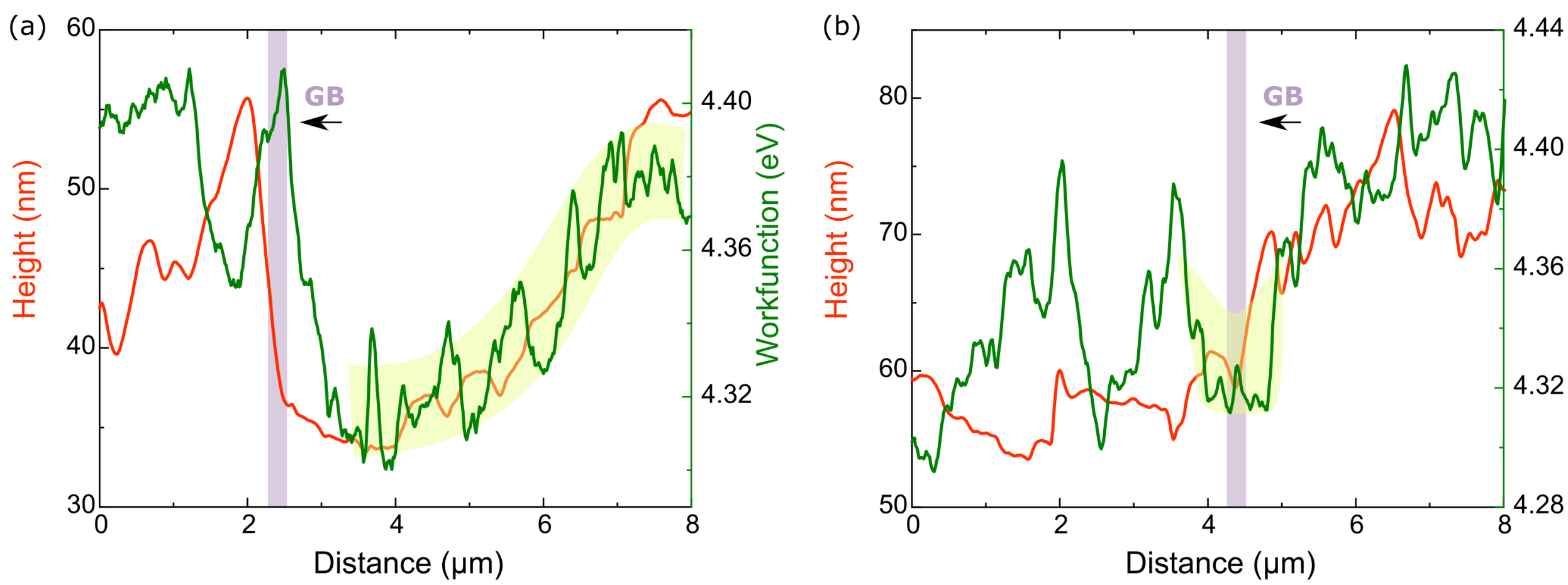

Figure S5. (a) Line profiles perpendicular to the grain boundary measured on the topography and KPFM image shown in Figure 2(b,e) of the main manuscript. (b) Similar profiles for the image are shown in Figure 2(c,f). The dashed lines are guides to the eye to highlight the gradual changes of the workfunction as a function of distance from the grain boundary. The vertical lines show the approximate position of the boundary.

S5 X-ray photoelectron spectroscopy (XPS) showing the removal of carbon contamination after the annealing procedure.

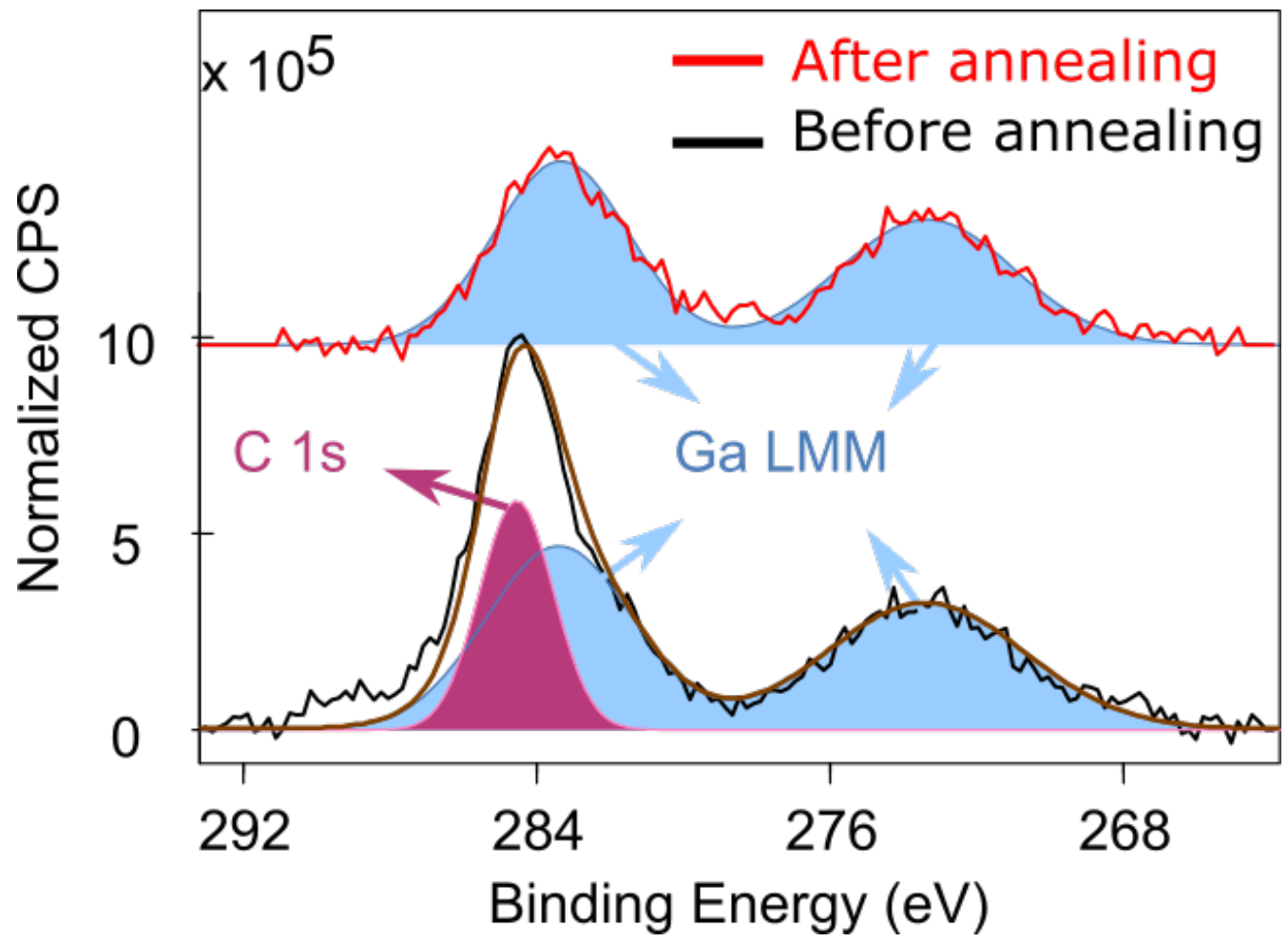

Figure S6. XPS high-resolution profiles before and after annealing the sample for 30 minutes at 200 °C. Deconvoluting the Ga LMM and from C1s peaks indicates the vanishing of residual carbon on the surface after annealing.

XPS was used to monitor compositional changes on the sample surface between each step of K-deposition. Figure S6 shows a high-resolution profile in the binding energies where the peak C1s from the element carbon is expected. Indeed, carbon and the Auger Ga LMM peaks were detected for the sample in the as-grown condition, depicted by the dark line profile at the bottom of the image. The C1s peak is highlighted in pink and the Ga LMM in blue. The residual carbon observed in the as-grown condition disappears after annealing the sample at 200°C for 30 minutes, as proven by the absence of the C1s peak in the upper line profile. Considering the detectable limit of the XPS, no other surface contamination was measured for the analyzed samples.

S6 Workfunction distributions during K deposition steps.

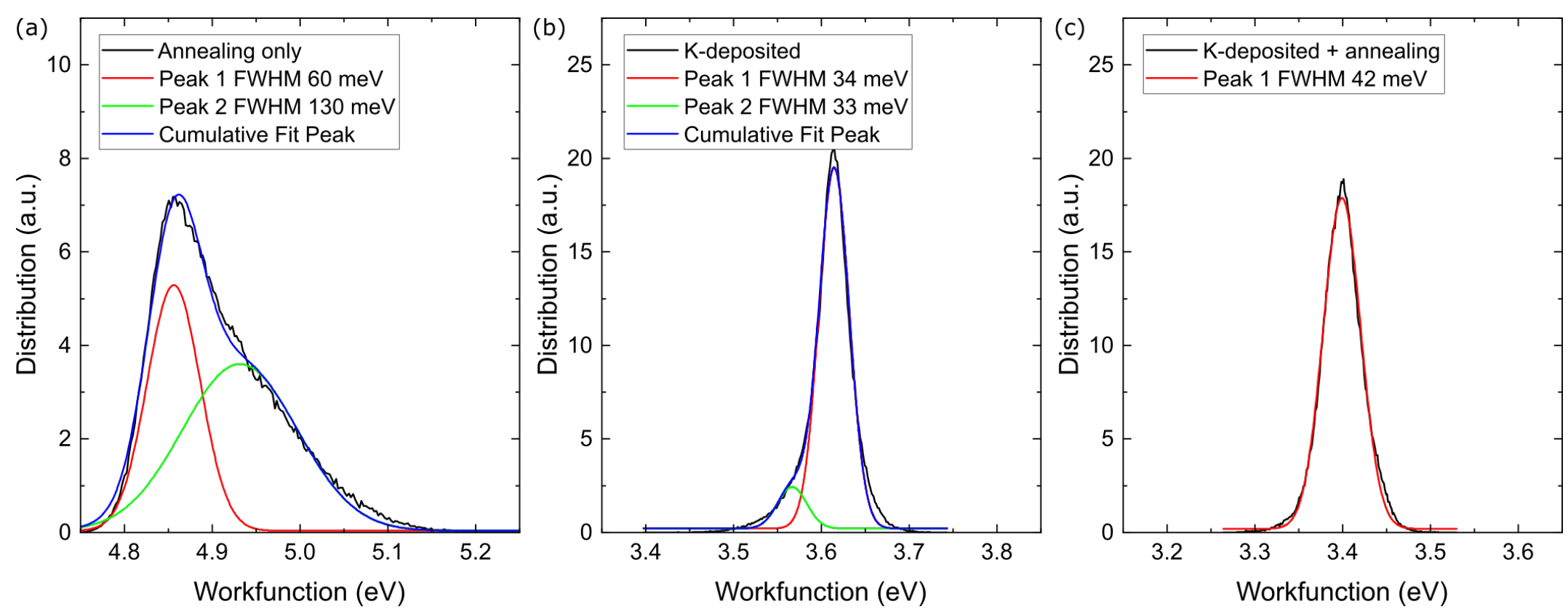

Figure S7. Workfunction distributions.

Figure S7 shows the workfunction distribution values for the following samples: annealed (without K), after K deposition, and after annealing (with K). The sample without K has two well-defined peaks in the distribution (Figure S7 (a)). These two peaks represent two different surface terminations: the lower workfunction values refer to the $\{001\}_{tet}$ facets, and the higher workfunction values refer to the $\{112\}_{tet}$ facets. The broader peak for the higher workfunction values is due to the presence of different surface terminations on the $\{112\}_{tet}$ facets (cationic and anionic), which lead to slightly different workfunction values. For the K-deposited cases (Figure S7 (b) and (c)), two peaks are less evident, which can be explained by the fact that the surface is now covered by a single element: K.

S7 Procedure for the extraction of the grain boundary workfunction variations

The following procedure was used to extract the grain boundary workfunction from the KPFM images. In Figure S8(a)-(c), the topography, the workfunction, and the error signal of the topography are shown. In all three images, the grain boundary is visible. The error signal is best suited to find the position of the grain boundary since here the contrast was best. To find the exact position of the grain boundary, a mask was drawn with a digital pen over the position of the grain boundary. The width was chosen such that the position was captured. This is shown in Figure S8.

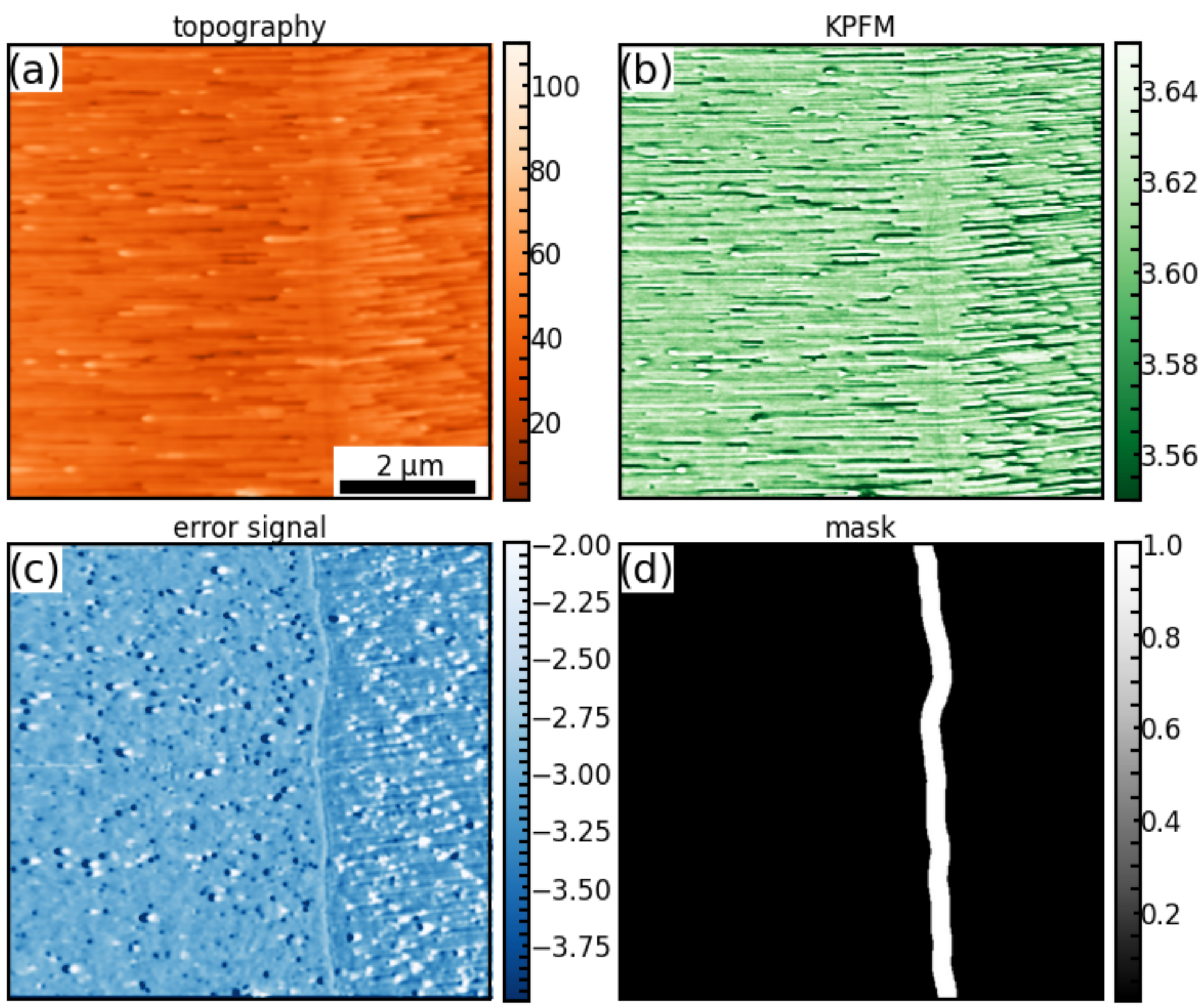

Figure S8. (a) topography, (b)workfunction map, and (c) error signal of the topography measured in the region shown in Figure 2c of the main manuscript. (d) masked grain boundary region

In the next step, each line of the image was analyzed. The region was confined to the masked region only. For each line, the position of the grain boundary was deduced from the maximum of the error signal. This was done with a Python script. This procedure was successful for at least 90% of the lines of the images. Some lines were not correctly identified. Therefore, the position of the grain boundary was plotted

and fitted with a polynomial function, resulting in a smooth curve. Finally, the resulting grain boundary was overlayed with the error signal, the topography, and the workfunction map as shown in Figure S9.

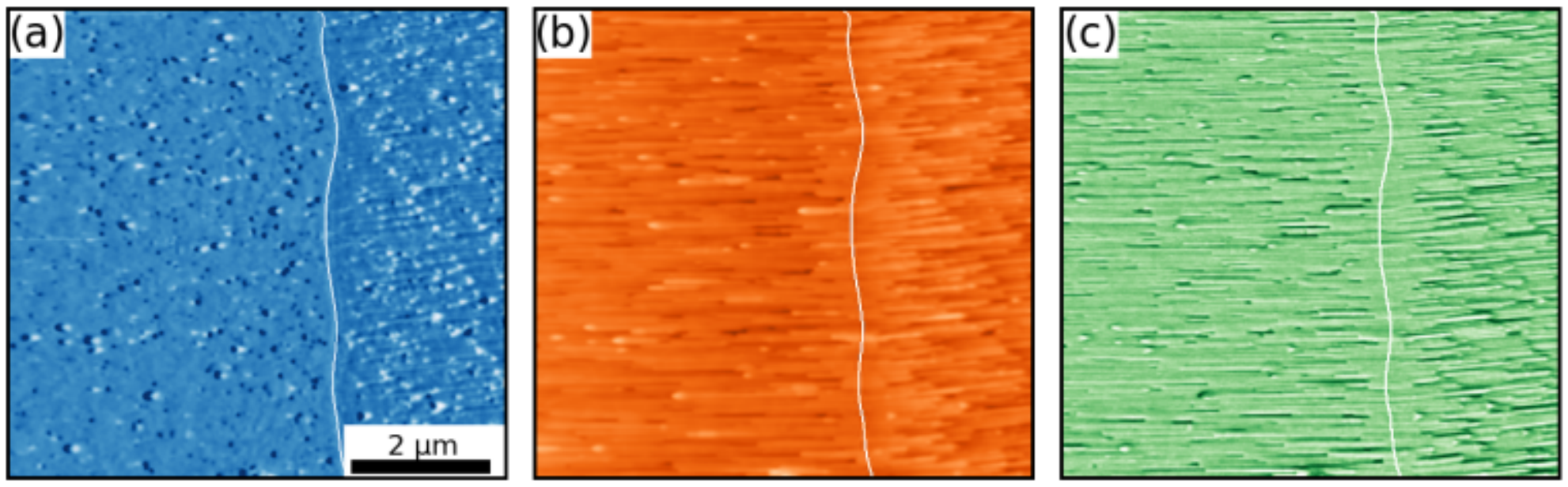

Figure S9. Position of the grain boundary highlighted in the error signal (a), the topography (b), and the workfunction map (c). For details, see the text.

After the determination of the grain boundary position, each line is averaged, taking the grain boundary as the center of the line. In the present case, the pixel resolution was 16 nm. In Figure S10, the result is shown for the case of the CIGSe sample after K deposition. The grain boundary is located at 320 nm, at slightly lower values than the minimum of the topography and of the workfunction, which was found at 340 nm. This makes sense considering that the error signal is largest when the topographic changes are largest. Consequently, the value is largest in the middle of the fact, left to the grain boundary.

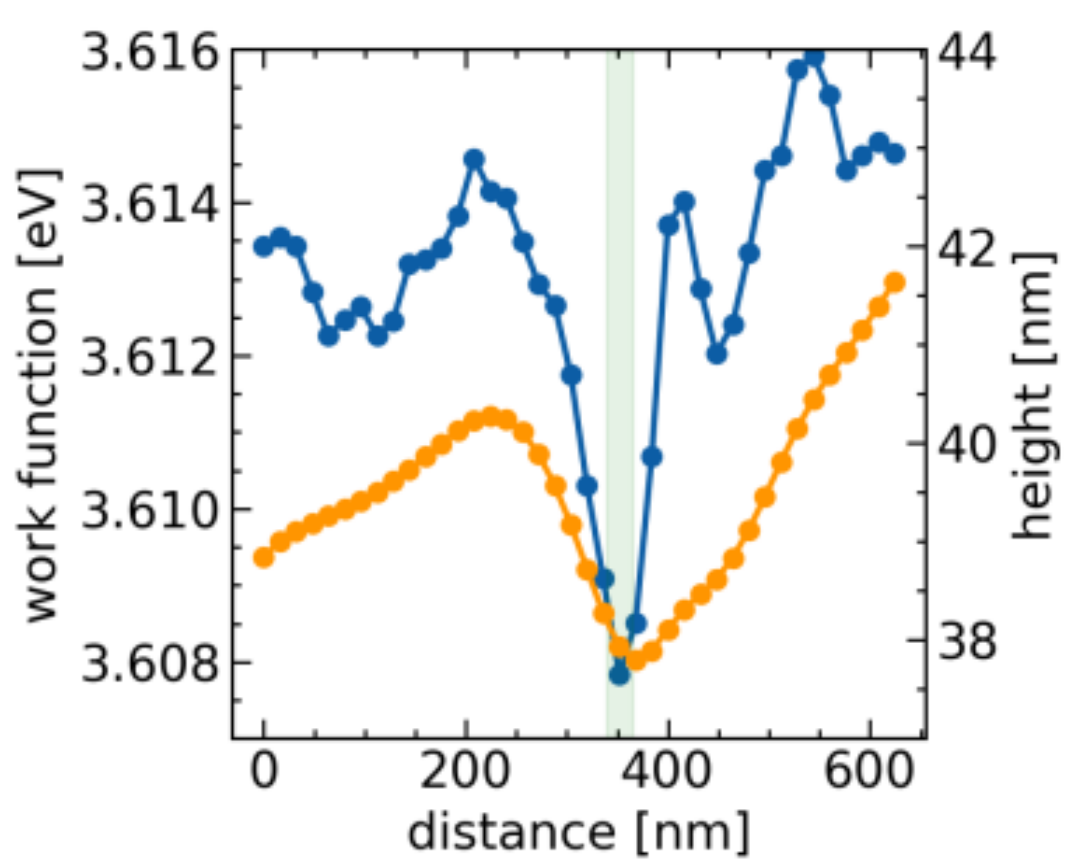

Figure S10. Resulting profiles of the workfunctions (blue) and topography (orange), at the position of the grain boundary, defined as the maximum in the error signal of the topography. The shaded region defines the estimated resolution of the tip, which is given by the tip apex size (here: 25 nm)

S8 Evaluation procedure: depth-dependent KPFM measurements

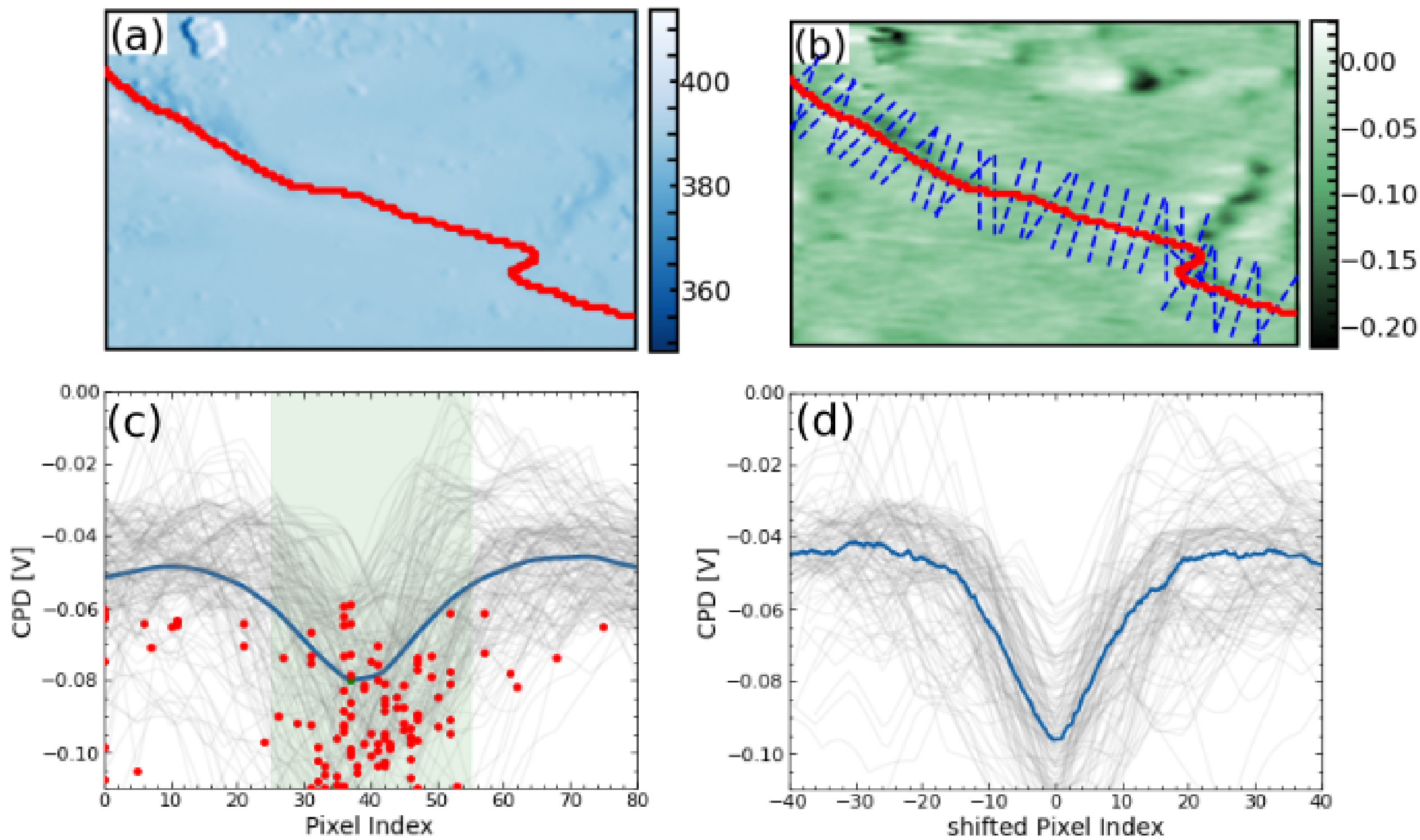

Figure S11. (a) error signal overlayed with an approximate position of the grain boundary. (b) KPFM measurement with the overlay of the grain boundary position and line profiles perpendicular to the grain boundary. (c) profiles deduced from the data shown in (b) (gray), together with the mean value shown in blue. The minimum as each line profile is highlighted with a red dot. Shifted line profiles according to the minimum of the workfunction.

In Figure S11(a), the error signal and the position of the estimated position of the GBs are shown. The position of the boundary was again highlighted with a digital pen in order to obtain a rough estimate. In contrast to the previous analysis, the grain boundary was not vertical, and therefore, for each pixel, a line profile perpendicular to the grain boundary was calculated. This is exemplarily shown for every 10th profile in Figure S11(b). Then each profile was plotted together with an average line profile in Figure S11(c). The minimum value of each workfunction profile is marked with a red dot. The minima are scattered, since their position has not yet been deduced correctly. The profiles were based on the mask, which was only approximate. Therefore, each line of workfunctions was shifted so that the minima of the workfunctions matched. Next, profiles where the detected minimum was more than 200 nm from the expected grain boundary position, were excluded from the analysis. This discrepancy in detecting the

minimaposition arose because the initial GB mask was based on the AFM error signal; however, since the topography in this region is highly uniform, the weak error signal led to some imprecision in the automated boundary detection. To ensure accuracy, we performed a visual inspection of the KPFM images, confirming that the profile minima correctly correspond to the GB location. Of the 158 line profiles initially collected, 121 were selected for the final analysis. Notably, the removal of these outliers had a minimal impact on the results, shifting the calculated band bending only slightly from 51 meV to 49 meV. The result of this correction is shown in Figure S11(d). The drop in the workfunction, already visible in the uncorrected average, was now resolved more clearly, and this curve was used in the main manuscript.

S9 SIMS additional information

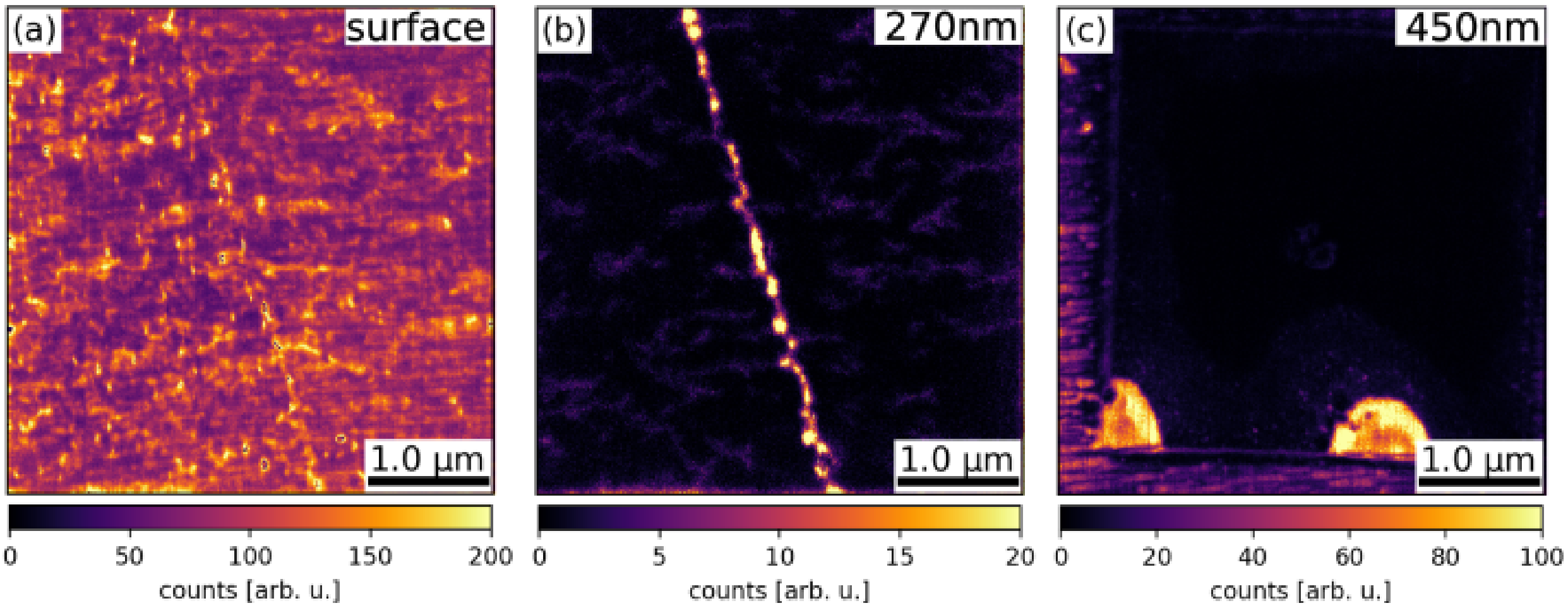

Figure S12. SIMS imaging (potassium) at the surface (a), at a depth of 270 nm (b), and at a depth of 450 nm (c).

In Figure S12(a), the K-signal in the HIM-SIMS is displayed. No material was removed prior to the deposition. After the removal of 270 nm (Figure S12(b)), only the grain boundary was visible in the SIMS imaging. Finally, after 450 nm Figure S12(c), the grain boundary contrast was no longer visible, and only some remains at the edges of the crater were visible.

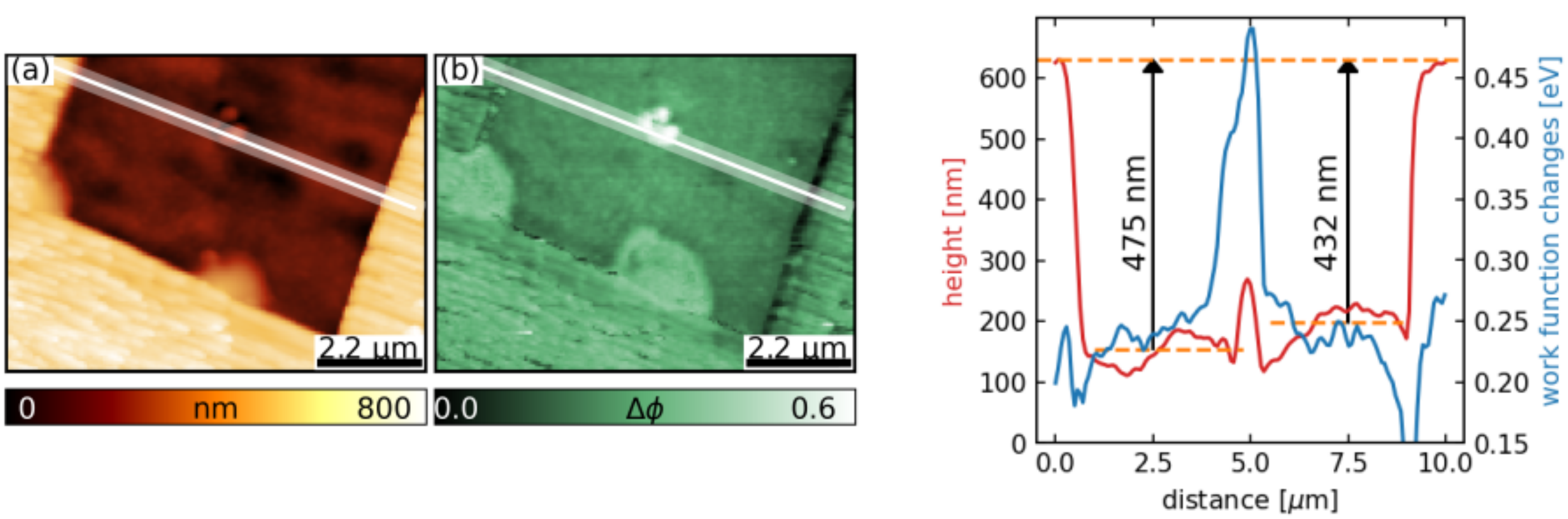

Figure S13. (a) AFM and (b) KPFM images in the same crater as shown in Figure S12(a-c). A line profile is shown in (c). The width of the line profile is shown as a shaded area in (a),(b).

This sample was subsequently measured in AFM, and the results are presented in Figure S13. The image is titled with respect to the SIMS imaging; however, the same region could be identified. On average, a value of 450 nm was found, which was used to calibrate all other depth profiles. The KPFM images

displayed regions where there was still some material left (in the corners of the crater), which exhibited a slightly larger workfunction than the rest of the crater. The measured crater depth was slightly different for the regions left and right of the grain boundary. It is important to point out that air exposure could not be avoided between the SIMS and this KPFM measurement; consequently, oxidation and contamination of the surface are anticipated, making this result a qualitative reference only.

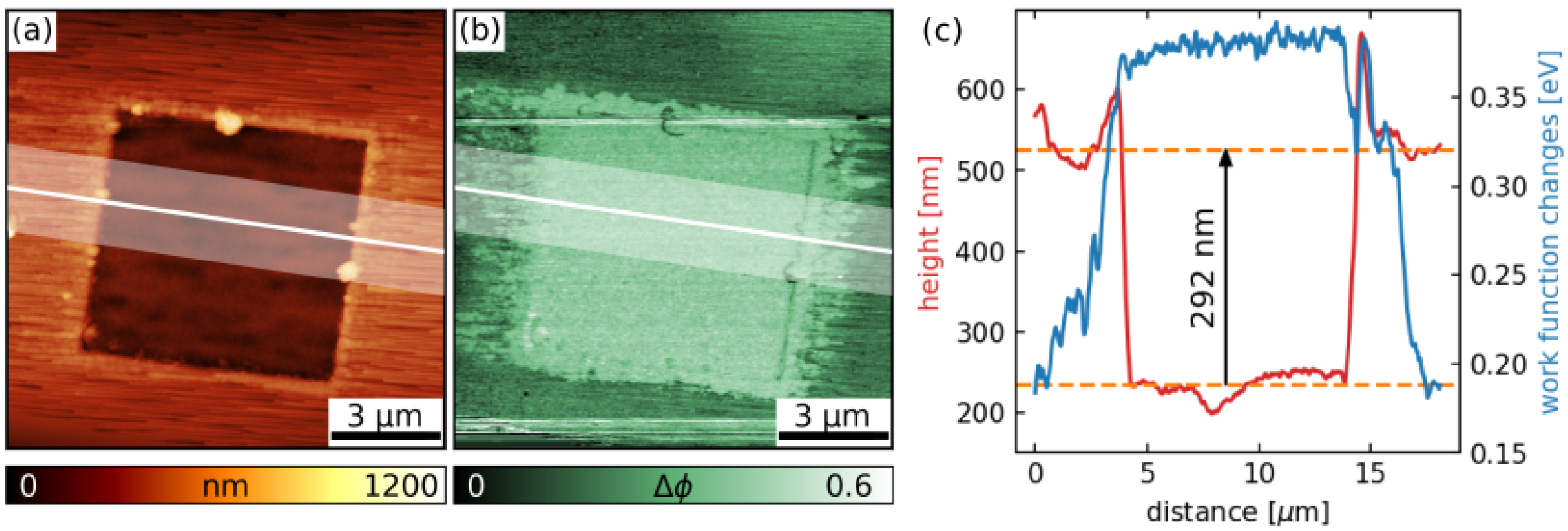

Figure S14. AFM, KPFM, and line profiles in another crater, where the depth profiling in SIMS was interrupted earlier. The workfunction is increased in the crater, compared to the surroundings, which is different from Figure S13.

Finally, another crater was also analyzed via KPFM in order to better understand the changes in workfunction as a function of depth. This crater was produced during the SIMS measurements, however, the measurement was interrupted after 300 nm of sputtering. In accordance with the depth-dependent KPFM measurements shown in the main manuscript, the workfunction was larger inside the CIGSe than on the surface. However, the values changed only by 200 meV compared to 500 meV for the measurement where the AFM tip was used for depth profiling. The difference is attributed to sputtering-induced damage and amorphization of the CIGSe, which is less drastic for AFM tomography.

S10 APT additional information

Local magnification effect artifacts have been claimed long ago to occur during APT experiments [1] when regions such as precipitates or grain boundaries with higher or lower evaporation field are present in the bulk material. Yet, there is an effective way to reduce the local magnification effect and that is to place the grain boundary perpendicular to the tip axis (z axis), since it had been demonstrated that the local magnification effect increases continuously as the grain boundary approaches an orientation parallel to the tip axis [2]. It is true that in the case of these very thin CIGS layers in the present work, most of the grain boundaries analysed (3 GBs investigated) were placed almost parallel to the z-axis due to their columnar growth orientation in the thin-film. The same goes also for the K-containing sample (4 GBs investigated), although here also one 45° inclined GB could also be analysed. If we compare both K-free and K-containing GBs with similar inclination (parallel to the z axis) and assuming that the local magnification effects are similar, we always observe a distinct difference in the width of the GBs between K-free and K-containing samples.

Moreover, similar alkali-free CIGS thin-films were investigated in two previous works [3,4] where the GBs had different inclinations from perpendicular to the tip axis to parallel to the tip axis. By comparing GBs with the same inclinations, the authors could observe similar trends: the GBs free of alkalis exhibit larger widths than those containing alkalis.

References SI

1. Miller, M.K. *et al.* Local magnification effects in the atom probe. *Surface Science* DOI: 10.1016/0039-6028(91)90449-3 (1991).

2. Blavette, D., *et al.* Atomic-scale APFIM and TEM investigation of grain boundary microchemistry in Astroloy nickel base superalloys. *Acta Materialia* DOI: 10.1016/S1359-6454(96)00087-0 (1996).

3. Cojocaru-Mirédin, O., *et al.* Grain Boundaries in Cu(In, Ga)Se_2: A Review of Composition–Electronic Property Relationships by Atom Probe Tomography and Correlative Microscopy. *Advanced Functional Materials* DOI: 10.1002/adfm.202103119 (2021).

4. Karami. A., *et al.* Sodium in Cu(In, Ga)Se2 Solar Cells: To Be or Not to Be Beneficial. *Solar RRL*,

DOI: 10.1002/solr.202300544 (2023).
